# Time Trends and Income Inequalities in Cancer Incidence and Cancer-Free Life Expectancy – a Cancer Site-Specific Analysis of German Health Insurance Data

**DOI:** 10.3389/fonc.2022.827028

**Published:** 2022-04-14

**Authors:** Fabian Tetzlaff, Jens Hoebel, Jelena Epping, Siegfried Geyer, Heiko Golpon, Juliane Tetzlaff

**Affiliations:** ^1^ Medical Sociology Unit, Hannover Medical School, Hanover, Germany; ^2^ Division of Social Determinants of Health, Robert Koch Institute, Berlin, Germany; ^3^ Comprehensive Cancer Center Hannover, Hannover Medical School, Hanover, Germany; ^4^ Department of Pneumology, Hannover Medical School, Hanover, Germany

**Keywords:** cancer incidence, time trend, income inequalities, compression of morbidity, Germany, cancer-free life expectancy

## Abstract

Cancer represents a major burden of morbidity and mortality globally. So far, however, little is known on time trends and inequalities in the lengths of life spent free of any cancer. This study steps into this gap by analyzing time trends and income inequalities in cancer-free life expectancy (CFLE). For this retrospective cohort study, data of a large German health insurer were used (N = 3,405,673individuals, 2006–2018). Income inequalities were assessed using individual income (<60% of German average income (GAI) and ≥60% of GAI). Trends in incidence risks were analysed employing proportional-hazard regression models by splitting the observation time into three periods of 52 months. Trends in CFLE in total and for the most common site-specific cancers were calculated based on multiple decrement life tables. Incidence rates declined in almost all cancers and CFLE increased substantially over time (49.1 (95% CI 48.8-49.4) to 51.9 (95% CI 51.6-52.2) years for men, 53.1 (95% CI 52.7-53.5) to 55.4 (95% CI 55.1-55.8) years for women at age 20 for total cancer) and income groups. Considerable income inequalities in cancer risks were evident in both sexes, but were more pronounced in men (total cancer HR 0.86 (95% CI 0.85-0.87)), with higher-income individuals having lower risks. The highest income inequalities were found in colon (HR 0.90 (95% CI 0.87-0.93)), stomach (HR 0.78 (95% CI 0.73-0.84)), and lung cancer (HR 0.58 (95% CI 0.56-0.60)) in men. A reverse gradient was found for skin (HR 1.39 (95% CI 1.30-1.47) men; HR 1.27 (95% CI 1.20-1.35) women) and prostate cancer (HR 1.13 (95% CI 1.11-1.15)). The proportion of CFLE in total life expectancy declined for lung, skin and cervical cancer in women, indicating a relative shortening of lifetime spent cancer-free. In contrast, increasing proportions were found in breast and prostate cancer. To our knowledge, this is the first study analysing trends and income inequalities in CFLE. The life span free of cancer increased clearly over time. However, not all cancer types contributed equally to this positive development. Income inequalities persisted or tended to widen, which underlines the need for increased public health efforts in socioeconomically vulnerable groups.

## Introduction

Cancer is a major burden of morbidity and mortality globally (1). In Germany, the leading cancers (lung, colon, breast, and prostate cancer) cause approximately 2 million years of life lost in 2017 (2). Despite this very high number of years of life lost, it is encouraging that incidence rates of most cancer types have either been declining or remained quite stable in the past decades. Exceptions to this development are lung cancer in women and skin cancer in both sexes, where the incidence among the German population tended to increase over time (3). Despite the high cancer burden overall, life expectancy has continued to rise over the past decades (4). However, current German studies report substantial social inequalities in life expectancy at individual level (5, 6) as well as in terms of regional disparities (7). Against the backdrop of rising total life expectancy on the one hand and high cancer burden on the other hand, the question arises whether these increases in life expectancy are accompanied by increases in cancer-free life expectancy (CFLE) and whether there are social inequalities in this development.

There is considerable evidence that health inequalities are primarily rooted in social inequalities. This has been shown in terms of both more general subjective health measures [e.g. self-rated health (8)] and more specific health indicators, e.g. substantial inequalities were found in objective outcomes such as cancer incidence and mortality [e.g (9, 10)]. However, little is known about time trends in social inequalities in healthy life years, or in our case life years free of cancer. Therefore, this study also aims to examine social inequality in CFLE and its development over time.

International studies have shown substantial social disparities in morbidity and mortality from common cancers, with individuals of low socioeconomic position having increased incidence risks [e.g (9–13)]. Moreover, the few studies analysing time trends in social inequality in total and site-specific cancer incidence reported rising inequalities over time (9, 10, 14). However, previous studies have shown that mainly lung cancer and colorectal cancer that account for increasing inequalities in total cancer mortality over time (9, 10, 15). For prostate cancer, female breast cancer, and skin cancer incidence a reverse social gradient was observed in several high-income countries, showing higher incidence rates in higher socioeconomic groups (9, 10, 16–19).

One of the rare studies, analysing inequalities in expected survival time after disease onset reported persisting educational inequalities in Sweden for colon, lung and female breast cancer between 1998 and 2017. In all three cancers, life years after initial diagnosis rose irrespective of educational status over time (20). However, this study focused on the average survival time after the onset of the cancer diagnosis in the population. The concept of “healthy life years” differs from this, as it describes the number of years of life without a specific health condition that can be expected based on the incidence and mortality rates currently observed in the respective population. To calculate CFLE, the transition rates of incidence, mortality without cancer as well as mortality after cancer diagnosis are needed. So far, the evidence on trends in health expectancies with respect to specific diseases is very limited. Studies based on health insurance data investigated this issue with focus on stroke, myocardial infarction, and lung cancer-free life expectancy (21–23).

While the incidence of almost all cancers in Germany is well documented by the Federal Health Monitoring System (3), there is a lack of evidence shedding more light on social inequalities in cancer incidence and mortality in Germany. In one of the few existing studies, Hoebel et al. analysed inequalities in cancer incidence based on a composite index of regional socioeconomic indicators of income, education and occupation at the German district level (11). For the cancers of the lung, stomach, kidney, bladder, and cervix uteri, the authors reported substantial inequalities for both sexes with individuals living in deprived areas having higher incidence rates. Furthermore, a reverse gradient for skin cancer was found indicating higher rates among the less deprived districts (11). To our knowledge, the only study based on individual data investigating time trends in inequalities in life years free of cancer and with cancer focused on lung cancer (21). This study reported rising income inequalities in life years free of lung cancer for both sexes and in life years affected by lung cancer in women. For both sexes, individuals with lower income are particularly disadvantaged since their gains in cancer-free life expectancy were much lower than among the high-income group. The study shows that the proportion of life years affected by lung cancer in total life expectancy decreased in men but increased among women over time (21). For other cancers, evidence for Germany is lacking.

To our knowledge, studies investigating time trends in CFLE and the corresponding trends in social inequalities are missing so far. The aim of our study is to analyse time trends in CFLE in the most common cancer sites (colon, lung, stomach, skin, prostate, breast, and cervix uteri) based on German health insurance data.

The study is guided by the following research questions:

1. How did site-specific cancer incidence develop over time?

2. What income inequalities exist in site-specific cancer incidence risks?

3. How did cancer-free life expectancy develop over time? Did the developments differ by income group?

4. How did the proportion of cancer-free life expectancy in total life expectancy develop over time? Did the developments differ by income group?

## Material and Methods

### Data

We used longitudinal data from 2005 to 2018 from one of the largest statutory health insurance funds in Germany, the *Allgemeine Ortskrankenkasse Niedersachsen* (AOKN). In Germany, it is compulsory for all residents to be insured with a health insurance fund (statutory or private). Approximately 90% of all German residents are insured with a statutory health insurance provider [e.g (5, 24).] and individuals can be insured regardless of employment status, occupation or age group. The AOKN insures about one third of the total population of the federal state of Lower Saxony. The data contain detailed information on morbidity (e.g. ICD-10GM diagnosis codes) as well as information on therapeutic procedures, socio-demographic information and mortality for the entire insurance population of approximately 3.4 million individuals in total [e.g (5, 24)]. For our analyses, we used the data of individuals aged 20 up to the oldest ages.

### Incidence

ICD-10-GM classification codes were used to define site-specific cancers in accordance with a previous study based on data from German cancer registries (11) (**Table 1**). For the analyses, it is important to distinguish between incident and prevalent cases in the data. For individuals who were already diagnosed at the beginning of the study period, the time of incidence cannot be identified. However, identifying the time of incidence is essential for calculating CFLE. Prevalent cases were therefore excluded from the analyses. To identify incident cases, the first inpatient or outpatient cancer diagnosis occurring in the individual insurance history was defined as incident diagnosis. We used one-year lookback periods to prevent prevalent cases to be incorrectly counted as incident cases. This implies in detail that the first individual cancer diagnosis must be preceded by an individual insurance history of at least one year without any other ICD-10GM cancer diagnosis. A previous methodical study has discussed this issue of selectivity since, given a 1-year lookback period, individuals with insurance periods shorter than 1 year have to be excluded from the analyses. These drop-outs from the study population could vary according to socioeconomic status. This selectivity increases with the length of lookback period applied. However, the study shows that the selectivity bias remains acceptable when lookback periods of 1 year are used (25). Furthermore, we additionally applied the minimum two quarter criterion to outpatient diagnoses, i.e. the first (outpatient) diagnosis has to be confirmed by a second outpatient or inpatient diagnosis in a second quarter of the respective year to be defined as incident. An exception was made if the person died within the quarter of diagnosis. This procedure is well-established to ensure the validity of incident cases from outpatient health claims data [e.g (26)]. To analyse time trends and to increase the number of incident cases by subgroup (sex, income group, single-year age group), the observation time of 13 years (2006 to 2018) was divided into three periods of 52 months. The year 2005 was only used as look-back period for the first year of the first period.

**Table 1:** Characteristics of the study population aged 20 and older: exposures in person-years, number of death cases, and number of incident cases by sex and time period.

		Men			Women		
		Period 1	Period 2	Period 3	Period 1	Period 2	Period 3
**Person-years at risk**	Total	2,810,704 (100%)	3,278,098 (100%)	3,487,981 (100%)	2,885,139 (100%	3,169,049 (100%)	3,406,880 (100%)
	Lower income	1,270,498 (45%)	1,410,850 (43%)	1,339,464 (38%)	2,195,213 (76%)	2,337,882 (74%)	2,281,654 (67%)
	Higher income	1,540,205 (55%)	1,867,248 (57%)	2,148,516 (62%)	689,927 (24%)	831,167 (26%)	1,125,226 (33%)
**Number of incident cases** **and** **crude** **mean age at incidence**	Total cancer (C00-C97)	39,184 (100%) 69 yr	40,885 (100%) 69 yr	36,765 (100%) 69 yr	39,924 (100%) 70 yr	39,468 (100%) 70 yr	36,601 (100%) 69 yr
	Colon cancer (C18-C20)	4,511 (12%) 71 yr	4,642 (11%) 71 yr	4,032 (11%) 71 yr	4,774 (12%) 75 yr	4,563 (11%) 75 yr	3,775 (10%) 75 yr
	Lung cancer (C33-C34)	4,981 (13%) 69 yr	5,210 (13%) 69 yr	4,809 (13%) 69 yr	2,006 (5%) 70 yr	2,406 (6%) 69 yr	2,428 (7%) 70 yr
	Stomach cancer (C16)	1,301 (3%) 70 yr	1,215 (3%) 70 yr	1,007 (3%) 69 yr	1,113 (3%) 75 yr	1,038 (3%) 73 yr	822 (2%) 73 yr
	Skin cancer (C43)	1,173 (2%) 65 yr	1,627 (4%) 65 yr	1,584 (4%) 65 yr	1,512 (4%) 65 yr	1,971 (5%) 64 yr	2,101 (6%) 63 yr
	Prostate cancer (C61)	9,294 (24%) 72 yr	8,893 (22%) 72 yr	7,419 (20%) 73 yr	–	–	–
	Breast cancer (C50)	–	–	–	11,386 (29%) 68 yr	10,971 (28%) 68 yr	9,567 (26%) 68 yr
	Cervix uteri (C53)	–	–	–	1,093 (2%) 59 yr	1,224 (3%) 56 yr	1,365 (4%) 55 yr
	Other cancer	17,924 (46%) 67 yr	19,298 (47%) 66 yr	17,914 (49%) 67 yr	18,040 (45%) 71 yr	17,295 (44%) 70 yr	16,543 (45%) 70 yr
**Number of deaths**	Total deaths	58,584 (100%)	58,016 (100%)	55,393 (100%)	79,397 (100%)	72,906 (100%)	67,328 (100%)
	Total cancer (C00-C97)	15,870 (27%)	15,213 (26%)	13,421 (24%)	14,223 (18%)	12,515 (17%)	11,143 (17%)
	Colon cancer (C18-C20)	1,667 (3%)	1,603 (3%)	1,390 (3%)	1,915 (2%)	1,619 (2%)	1,296 (2%)
	Lung cancer (C33-C34)	3,842 (6%)	3,829 (6%)	3,409 (6%)	1,499 (2%)	1,691 (2%)	1,603 (2%)
	Stomach cancer (C16)	775 (1%)	636 (1%)	537 (1%)	672 (1%)	523 (1%)	422 (1%)
	Skin cancer (C43)	183 (0.3%)	186 (0.3%)	174 (0.3%)	231 (0.3%)	185 (0.3%)	185 (0.3%)
	Prostate cancer (C61)	1,783 (3%)	1,640 (3%)	1,324 (2%)	–	–	–
	Breast cancer (C50)	–	–	–	2,009 (3%)	1,711 (2%)	1,434 (2%)
	Cervix uteri (C53)	–	–	–	246 (0.3%)	195 (0.3%)	182 (0.3%)
	Other cancer	7,620 (13%)	7,319 (13%)	6,587 (12%)	7,651 (10%)	6,591 (9%)	6,021 (9%)

Period 1 (January 2006 to April 2010), period 2 (May 2010 to August 2014), period 3 (September 2014 to December 2018); percentages refer always to the respective total of person-years at risk, number of deaths, and the number of incident cases by period; yr ; years.

### Income Definition

In Germany, insurance fees depend on individual pre-tax income. Therefore, the insurance database contains individual income (e.g. salaries, pensions, social security payments). To ensure comparability over time, we adjusted income for inflation by using the same resource driven approach as in previous studies [e.g (5, 21)]. As in previous studies (21, 22), the German average income from salaries (GAI) in 2006 [published in the appendix 1, Book VI of the Social Code (SGB VI. Anlage 1. https://www.gesetze-im-internet.de/sgb_6/anlage_1.html)] was used as cut-off value to define income groups. However, due to low numbers of cancer-site specific incident cases in single-year age groups (e.g. cervix uteri), it was necessary to categorise individual income into two income groups: lower income (less than 60% of GAI) and higher income (≥ 60% of GAI). Subjects with missing income information were excluded from the analyses. Sensitivity analyses in a previous study showed that this proportion is highest among the younger age groups. However, the analyses also showed that excluding the income missings had only a minor effect on the level and trends in total life expectancy [see (5), Supplemental Material Tables A1, A4- A6].

### Statistical Analysis

First, we employed proportional hazard regression models with constant baseline hazards to estimate smoothed age-specific incidence and mortality rates from our data. The lifespan free of cancer depends on two competing hazard rates, namely the age-specific cancer incidence rate and the mortality rate among the cancer-free population. For both transitions, the time-to-event must be determined in order to estimate the hazard rates from regression model. For the transition to incidence, the time-to-event for each period results from the start of the individual observation (usually the start of the respective period for long-term insured individuals) and the time of incidence, or if no incidence occurs, the end of the individual observation time within the respective period (usually the end of the period). In a similar way, the time-to-death was calculated for each period as the time between the individual’s entry and the time of death or, alternatively, the end of the period or the end of the individual observation period (in the case of an earlier exit from the insurance). For both transitions, the observation was censored at the time of the competing event. In the case of incidence, the hazard rates estimated from the regression models reported in the following are referred to as “incidence rates” giving them a straightforward interpretation.

The models were stratified by sex, income group, and period and include single-year age groups as second-degree polynomial. From these models, the smoothed age-specific hazard rates were estimated using the STATA post-estimation command “PREDICT, ha”. In the case of incidence, the hazard rates estimated from the regression models reported in the following are referred to as “incidence rates” giving them a straightforward interpretation. The incidence rate is reported per 100,000 person-years. In order to estimate general income inequalities and the time trends in incidence risks (referred to as “cancer risks” in the following) within income groups, the period data sets were combined and analysed separately for sex and cancer site. In a second step, the smoothed incidence and mortality rates were used as input to calculate the expected number of cancer-free life expectancy from multiple decrement period life tables. CFLE depict the expected number of life years without any cancer at a given age x, assuming that the age-specific incidence and mortality rates with and without cancer of the respective period apply over the entire life course. This means that the interpretation of CFLE is very close to that of general life expectancy as it represents the subset of life expectancy spent free of any cancer. The methodological approach to determine multiple decrement life tables is based on Palloni (27). Using the total and income-specific life expectancies based on the mortality rates observed in the insurance data, we estimated the proportion of CFLE in total life expectancy (i.e. a proportion of 100% would indicate that all remaining life years are cancer-free). 95% confidence intervals were calculated from 1000 bootstrap replication. The statistical analyses were carried out with STATA 17 (28) and R4.1.2 (29). The graphical visualisation of the results was done in R (using the package “tidyverse”).

## Results

**Table 1** displays person-years at risk, the number of deaths, and incident cancer cases across the three observation periods. The data include approximately 3 million person-years per period in both sexes. Over time, 24-27% of the total number of deaths in men and 17-18% in women occurred after a cancer diagnosis. Except for lung and cervical cancer in women and skin cancer in both sexes, the absolute number of incident cases decreased for all other cancer sites from period 1 to period 3 (**Table 1**).

### Time Trends in Age-Specific Cancer Incidence

**Figure 1:**
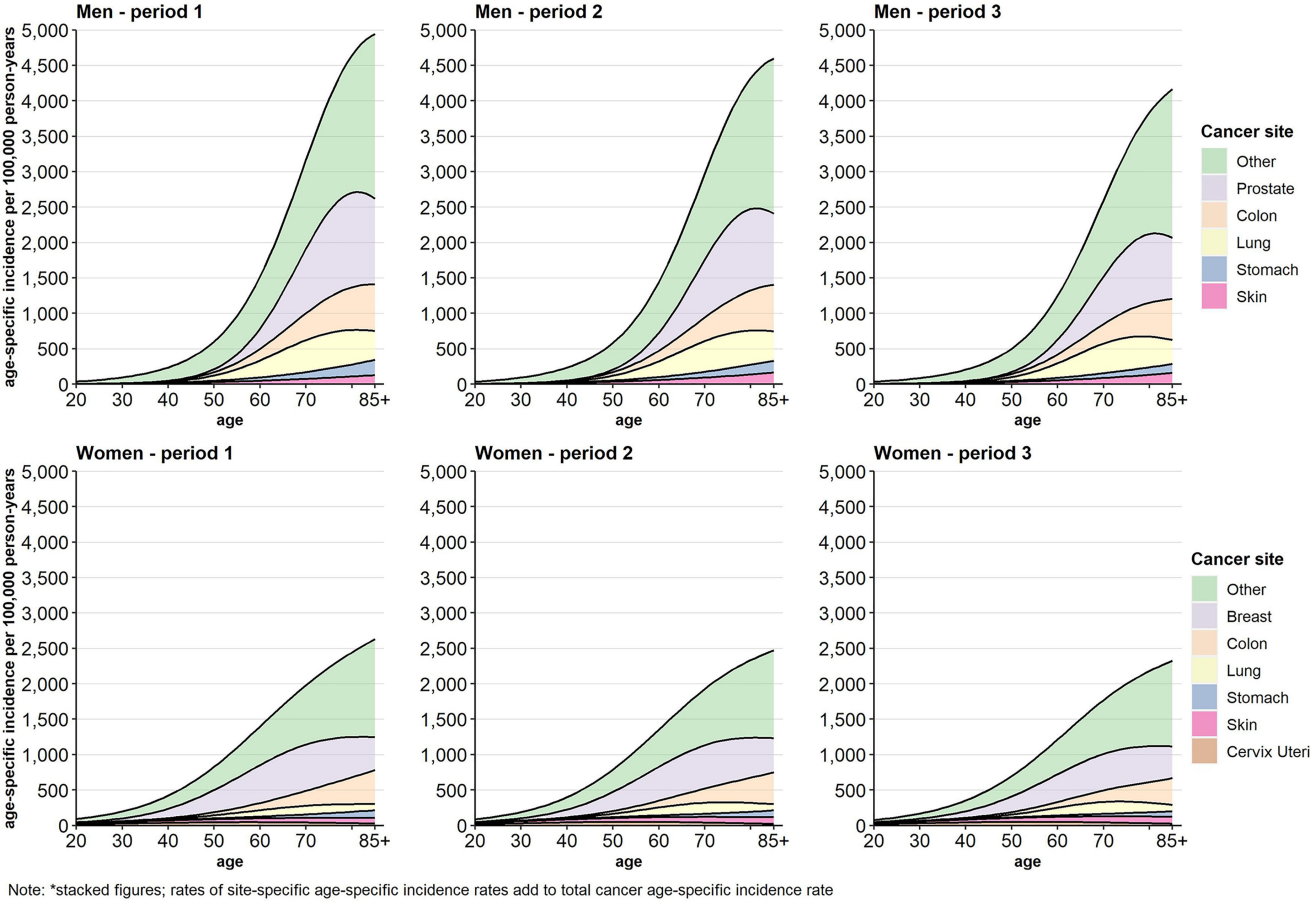
Age-specific incidence (stacked figure)* per 100,000 person-years by cancer site, sex, and period.

**Figure 1** displays the stacked cancer site-specific incidence rates by sex and time period across age. The cancer types are hierarchically stacked according to the level of cancer site-specific incidence rates in period 1. The single cancer site-specific incidence rates cumulate to the age-specific incidence rate of total cancer. In women as well as in men the incidences decreased over time and were postponed into higher ages (**Figure 1**). The contribution of the single cancer types to the development of the total cancer incidence varies over time as well as across age, sex, and income groups (**Figures S1–S7**, **Supplementary Material**). While the incidence rates for cervix uteri are highest between age 50 and 60, the mean age of incidence is beyond the age of 70 in all other analysed cancer sites. Except for lung cancer in women and skin cancer in both sexes where age-specific incidence rates tend to increase, age-specific incidence rates decreased in all other cancer types over time.

### Time Trends and Income Inequalities in Cancer Risks

**Figure 2:**
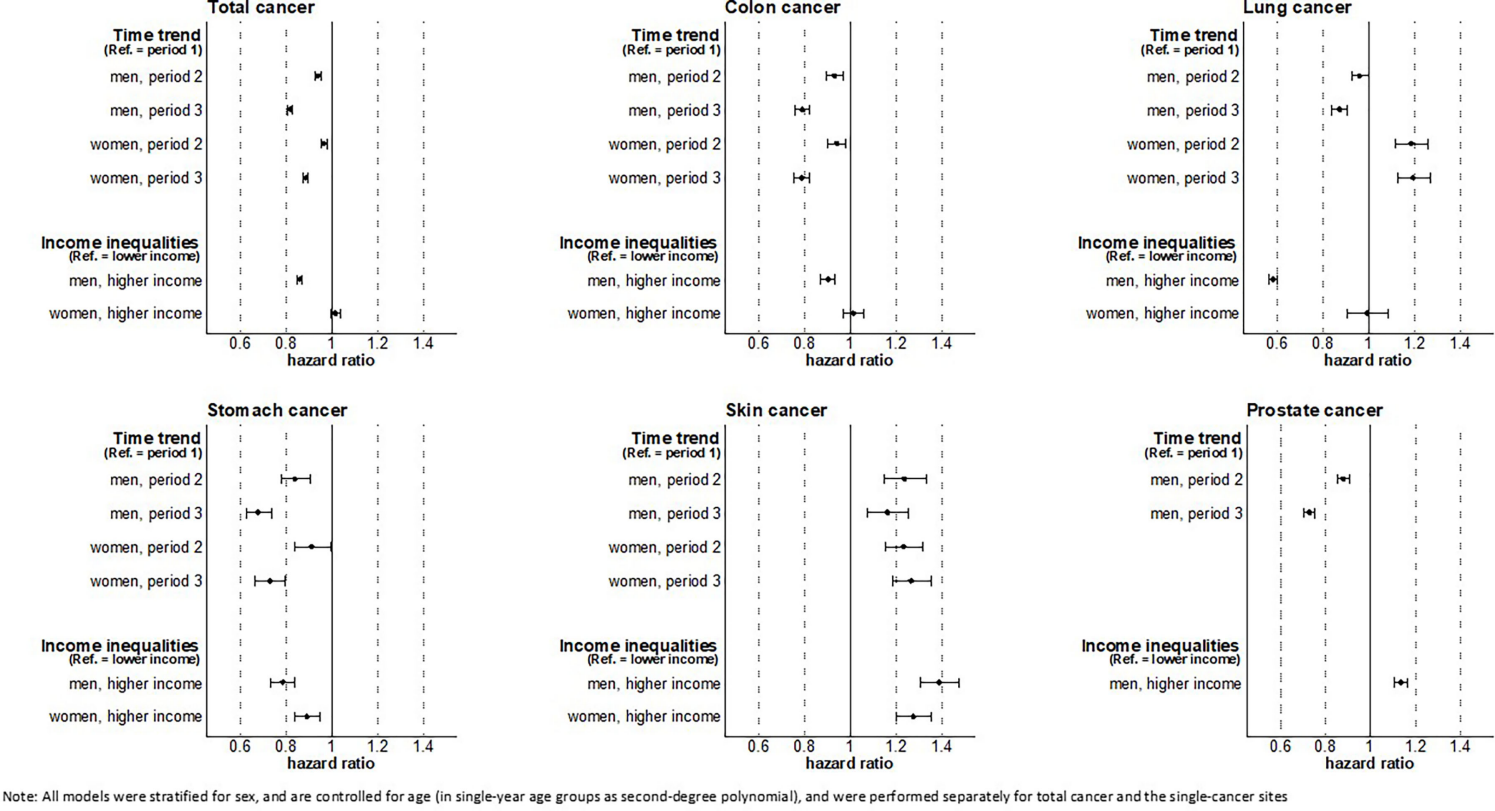
Time trend and overall income inequality in cancer risk in men and women.

Over time, decreasing risks of total cancer were found for men as well as for women. With respect to cancer site, risks decreased for colon, stomach, and other cancers in both sexes, for prostate and lung cancer in men, and for breast cancer in women. Increasing cancer risks were found for lung cancer in women and for skin cancer in both sexes (**Figure 2**).

The analyses show substantial income inequalities in the risk of total (HR 0.86, 95% CI 0.85-0.87), colon (HR 0.90, 95% CI 0.87-0.93), lung (HR 0.58, 95% CI 0.56-0.60), stomach (HR 0.78, 95% CI 0.73-0.84), and other cancer (HR 0.80, 95% CI 0.78-0.82) in men as well as in stomach cancer (HR 0.89, 95% CI 0.81-0.97), other cancer (HR 0.93, 95% CI 0.91-0.95), and cervical cancer (HR 0.85, 95% CI 0.79-0.91) in women. In these cancers, we found that individuals with higher income had lower cancer risks compared to individuals with lower income. In contrast, a reverse pattern of income inequality in incident cancers was found for prostate (HR 1.13, 95% CI 1.11-1.15) and skin cancer (HR 1.39, 95% CI 1.30-1.47 in men; HR 1.27, 95% CI 1.20-1.35 in women) in both sexes. No clear income inequalities were found among women for total, colon, lung, and breast cancer (**Figure 2**).

Focusing on the developments within income groups over time (**Figure S8**, **Supplementary Material**), we found similar trends in all cancer sites considered. In accordance with the general trend, risks of lung cancer in women (HR 1.22 in the lower-, HR 1.11 in the higher-income group) and skin cancer in both sexes increased in both income groups (HR 1.16 in lower- and HR 1.13 in higher-income men, HR 1.20 in lower- and HR 1.35 in higher-income women). For all other cancers, the cancer risks decreased substantially in both income groups over time. Overall, income inequalities persisted and changes over time remained limited. For all cancers combined, however, income inequalities slightly tended to widen in men, which may be driven by slight increases in income inequalities in some common cancers among men, e.g. prostate cancer (HR 0.74 in the lower- vs. HR 0.71 in the higher-income group) and lung cancer (HR 0.90 in the lower- vs. HR 0.88 in the higher-income group). Although income inequalities in total cancer incidence in women remained largely unchanged, a tendency towards increasing income inequalities were found in colon cancer, lung cancer, breast cancer and other cancer (**Figure S8**, **Supplemental Material**).

### Time Trends in Cancer-Free Life Expectancy

**Figure 3:**
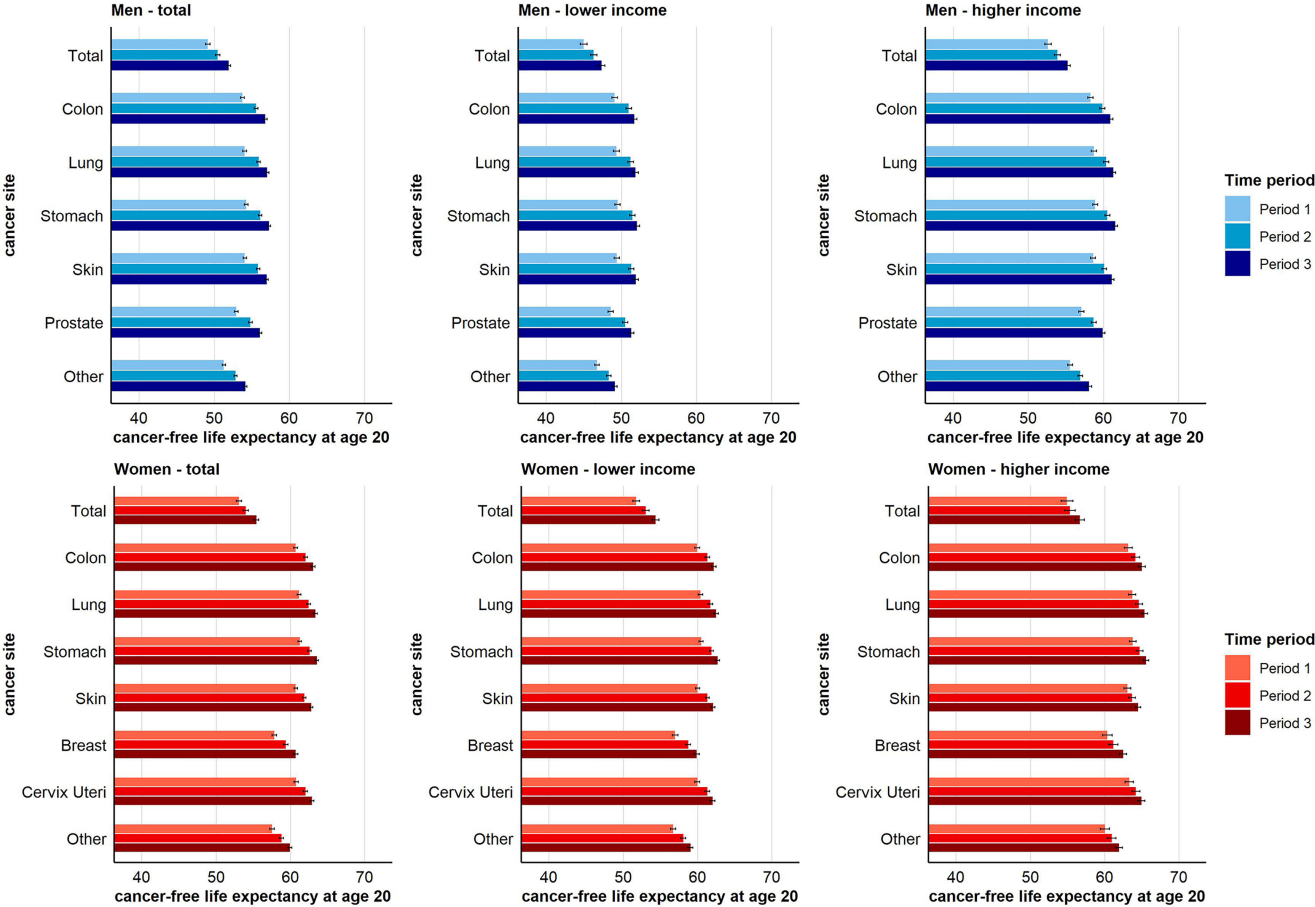
Time trend in cancer-free life expectancy for total and site-specific cancer by sex and income group at age 20 (955-CI).

For total cancer and all other cancer sites considered, CFLE in absolute terms increased over time. For total cancer, CFLE at age 20 increased from 49.1 (95% CI 48.8-49.4) to 51.9 (95% CI 51.6-52.2) years for men and from 53.1 (95% CI 52.7-53.5) to 55.4 (95% CI 55.1-55.8) years for women (**Figure 3**). However, individuals with higher income can expect substantially more CFLE than individuals with lower income. Nevertheless, for women, the gains in CFLE in most cancer sites are higher in the lower income group, which reduced the income inequalities in CFLE (e.g. in terms of total cancer at age 20 51.8 (95% CI 51.3-52.2) to 54.4 (95% CI 53.9-54.8) years in the lower income group vs. 54.9 (95% CI 54.2-55.8) to 56.7 (95% CI 56.0-57.3) years in the higher income group) (**Figure 3**). Among men, on the other hand, both income groups benefitted equally from the increase in CFLE (e.g. +Δ2.4 years in higher-income vs. + Δ2.2 years in low-income men for total cancer at age 20). Thus, income inequalities in CFLE in men remained stable over time (**Figure 3**). **Figures S9–S15** show the time trends in CFLE across age. The results indicate that increases in CFLE are visible across the full age range from age 20 up to age 85+ (**Figures S9–S15**, **Supplementary Material**).

**Figure 4:**
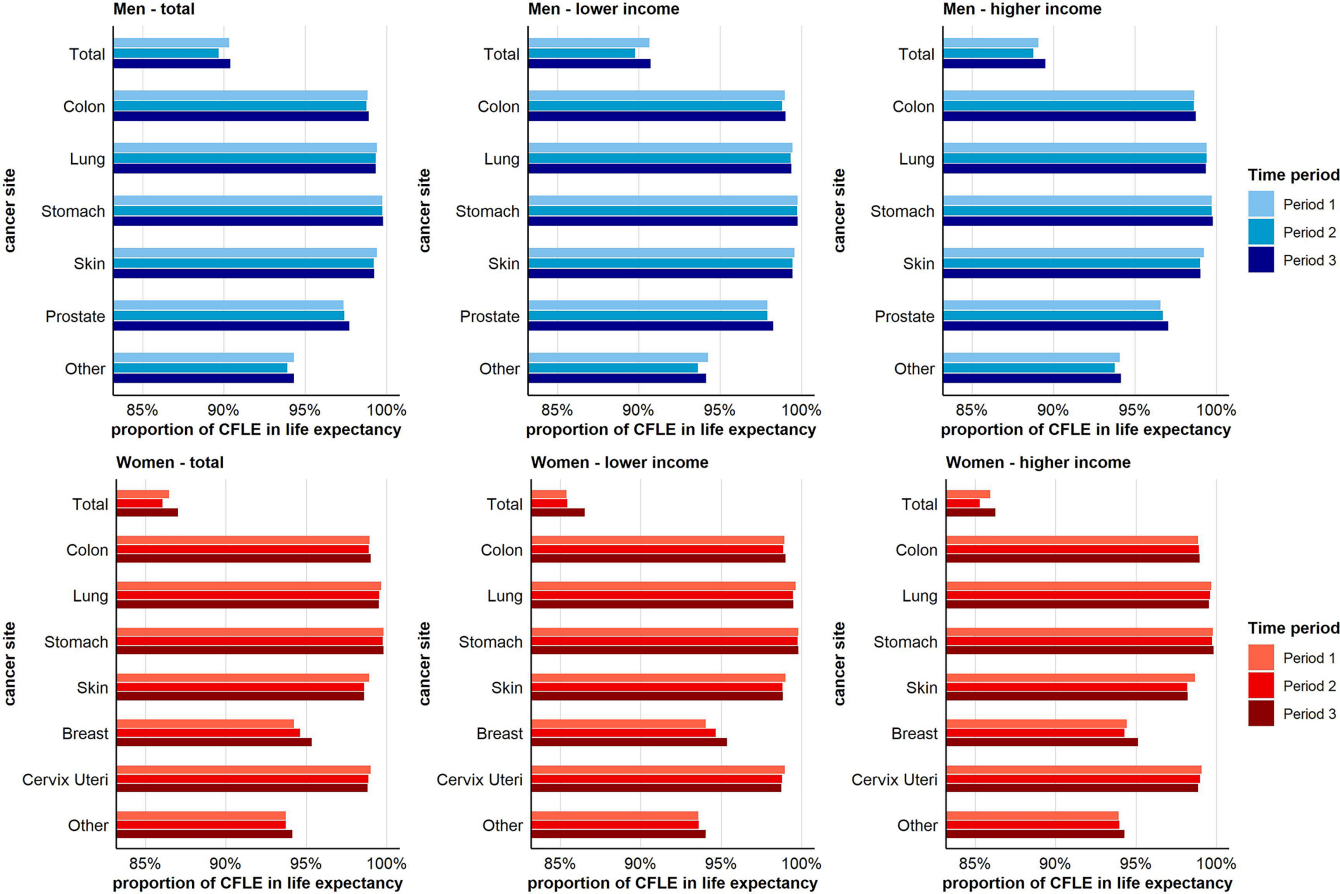
Time trend in the proportion (in %) of cancer-free life expectancy in total life expectancy for total and site-specific cancer by sex and income group at age 20.

**Figure 4** depicts the proportion of CFLE in the total life expectancy at age 20. The reader may note that the lowest proportion of CFLE across the different sub-figures is always found for total cancer. This is because the different site-specific cancer rates cumulate to the total cancer incidence rate. Consequently, CFLE in terms of total cancer are lower than those in terms of single-cancer sites. Combined for both income groups, we found no gains in the proportion of CFLE with respect to total cancer in men over time. This holds also for men with lower income. In men with higher income, gains in CFLE tended to be higher than increases in total life expectancy, leading to a higher proportion of CFLE in period 3 compared to period 1. With respect to skin cancer in men, for which a reverse pattern of income inequalities in cancer risks was observed (**Figure 2**), we found decreasing proportions of CFLE. This holds for both income groups, but was more pronounced among men with higher income. The clearest increases were found with respect to prostate cancer for which the proportion of CFLE increased in both income groups, though again at a faster pace among men with higher income (**Figure 4**).

In women, the proportion of CFLE tended to increase across periods. This increase was mainly driven by the increases in CFLE in breast and other cancers, with gains being higher among women with low income. In contrast, decreasing proportions were found for skin cancer, cervical cancer, and lung cancer, with decreases for cervix uteri and skin cancer being stronger in the higher-income group (**Figure 4**).

## Discussion

### Main Findings

To our knowledge, this is the first study examining health expectancies in terms of cancer as well as the corresponding social inequalities. The study is based on a large health insurance dataset, which allowed us to distinguish between different cancer sites and to take a closer look at the social epidemiological trends in incidence and the development of cancer-free life lifespan over time. Our analyses revealed a substantial decline in cancer incidence over time for almost all cancer types studied, with the exception of lung cancer and cervical cancer in women and skin cancer in both sexes, where increasing or stable trends were observed. These mostly positive developments also had a positive effect on CFLE. In all analysed cancer types, we found clear gains in CFLE indicating that the average life span spent free of cancer increased over time. This equally holds for men and women with lower and higher income respectively. However, the number of CFLE differs substantially between income groups due to differences in mortality and cancer incidence. Considerable income inequalities in cancer risk were found, especially among men disadvantaging individuals with lower income in most cancer sites (colon, lung, stomach). For women, the income inequalities are weaker but still evident in most types. A reverse gradient was found in prostate cancer and in skin cancer for both sexes. Overall, income inequalities in cancer risks persisted throughout the study period and time trends did not differ substantially between income groups. While CFLE clearly increased, we found increasing relative proportions of CFLE only among men with higher income in terms of total cancer and in terms of prostate cancer for both income groups. Both, the reduction in the incidence and the decreases in mortality contributed to this development. Decreasing proportions were found for skin cancer (both sexes) as well as for cervix uteri and lung cancer (women) since incidence and CFLE increased at a slower pace than life expectancy. For female breast cancer, in contrast, the proportion of CFLE clearly increased over time.

### Discussion With Previous Research

Previous studies revealed that a non-negligible proportion of the years of life lost were due to cancer. The considerable social inequalities in cancer incidence contributed to social increasing inequalities in mortality and widening differences in life expectancy between socioeconomic groups over time (2, 9, 30–33). These previous findings underline the high public health relevance of socioeconomically differentiated analyses of cancer morbidity and mortality. Decomposing the effect of different causes of death on gains in life expectancy, Doblhammer et al. have shown that a reduction in cancer mortality in Germany to the lower level of Sweden would increase life expectancy by about 0.7 years. The highest contribution of 0.4 life years can be attributed to lung cancer (34). Overall, the evidence on social inequalities in cancer morbidity and mortality for Germany is limited, especially with respect to time trends. However, our results on social inequalities are largely in line with former findings from a single-period analysis based on official cancer registry data (11). This study reported inequalities in cancer incidence using a regional index of socioeconomic deprivation. Despite measures of social inequalities differ, both studies reported considerable social inequalities in stomach and skin cancer in both sexes and in lung and colon cancer among males (11). In addition, our study provides a more detailed picture of the development of social inequalities in cancer incidence, mortality and CFLE over time, as we were able to perform time-trend survival analyses using data from individual insurance histories. In a previous study, this approach allowed us to get evidence of a reversing social gradient for lung cancer among older women in Germany, with higher incidence rates shifting from privileged to disadvantaged socioeconomic groups (9, 10, 14). This change is discussed as a consequence of the progressing “smoking epidemic”, which do not affect all population subgroups of a country simultaneously (35, 36). However, without taking into account the development in incidence rates over time, such changes in social inequalities remain masked. Our results on lung cancer in women illustrate that time trend analyses are preferable to single-period analyses whenever the dataset allows for such analyses (14).

In contrast to the regression analyses performed in this study, a wide body of international studies report a reverse gradient in female breast cancer for several high-income countries (9, 10, 16–18). Hoebel et al. also found a reverse gradient in female breast cancer in Germany (11). The absence of clear social inequalities in female breast cancer in our study may be explained by the development of income inequalities in the age-specific incidence rates over time. On the one hand, the analysis of the age-specific incidence rates (**Figure S6**) reveals increased rates among women up to their 60s with lower income in period 1. On the other hand, in the third period, women with higher income had a higher incidence compared to women with lower income, especially at age 60 and above where breast cancer occurs most often. The difference between income groups among younger and middle aged women disappeared over time as incidence rates decreased faster in the low-income group. Due to this development, incidence rates tended to be higher in women with higher- compared to lower-income, with clear differences emerging at age 60 and above. If this trend continues, it is therefore possible that a reverse gradient in cancer risk will also emerge in our AOKN study population in the future. We also observed similar dynamic processes in the age-specific incidence of lung cancer among women. Although we used a broader definition to operationalise lung cancer, the reported time trends in overall and age-specific income inequalities were similar to previous studies (14, 21).

The hypothesis of morbidity compression formulated by Fries in the 1980s postulates that the healthy lifespan increases over time (37). Fries considers advances in medicine and prevention to be primarily responsible for this increase (37). According to this, not only early detection and improved treatment of conditions such as cancer contribute to a reduction in mortality. Improved health literacy and primary prevention also contribute to the reduction of many chronic diseases over time. However, the development of average disease-free life span should be assessed against the backdrop of developments in total life expectancy. Therefore, not only absolute gains in disease-free lifespan should be considered, but also the development of disease-free lifespan in relation to the development of total life expectancy. Taking this into account, our results for female breast cancer as well as for prostate cancer, and less pronounced also for total cancer, are consistent with Fries’ hypothesis on morbidity compression.

A large number of studies suggest that social inequalities in behavioural, occupational, and environmental risk factors are major drivers of social inequalities in cancer incidence. In particular, smoking [e.g (38–41)], nutritional behaviour [e.g. high-calorie diet or alcohol consumption (3, 40, 42)], high uv-radiation exposure (3, 19), deprived housing and high levels of respiratory air pollution at the workplace [e.g. mould, radon, or nitrogen dioxide (43–45)] are known to foster the risk of various types of cancer. Therefore, it can be assumed that the reduction of behavioural and environmental risk factors has the potential to contribute substantially to further increases in CFLE in the future.

In addition, the earliest possible detection of tumours, e.g. within the framework of cancer screening programs, contributes to a reduction in stage at diagnosis and thus to a reduction in cancer mortality [e.g (46, 47)]. Between 2005 and 2009, a national programme for early cancer detection has been introduced into the statutory health care system in Germany, which expanded early detection services for the insured individuals. The national programme includes secondary prevention services such as the Papanicolaou-Test (PAP-test, from age 20, annually), palpation (from age 30, annually) and mammography (age 50-69, biannually) for women. For both sexes, the additional services include skin cancer screenings (from age 35, biannually), hemoccult test (age 50-54, annually; biannually from age 55) and colonoscopy (age 55 and again 10 years later) (48).

However, international research reported social differences in the utilisation of screening programmes, with individuals with lower socioeconomic status making less use of them [e.g (49, 50)]. This also holds true for Germany, leading to tumours being detected less frequently at an early stage in individuals with low social-economic status [e.g (48, 51, 52)]. Since 2007, girls and boys aged 11 can vaccinated against the human papillomaviruses (HPV) in Germany. With unrestricted access, it might be assumed that HPV vaccination has the potential to reduce social inequalities in cervical cancer. However, previous studies indicate that only approximately 50% of eligible girls and boys in Germany have received full vaccination protection (53). Furthermore, a systematic review by Murfin et al. indicates that both women with higher educational attainment and higher income are much more likely to be vaccinated against HPV (50). These inequalities in vaccination rates suggest that social inequalities in cervical cancer will persist or may even increase in the future. However, this could not been examined in our study, as the short period of time since the introduction of vaccination does not allow for conclusions on the impact of vaccination on incidence and mortality of cervical cancer at population level.

The substantial income inequalities in CFLE reported in this study as well as the well-known social inequalities in mortality underline the high importance of interventions aimed at reducing social inequalities in risk exposure and in participation in preventive screening and vaccination programs.

### Strengths and Limitations

We used data of a large German statutory health insurance provider with approximately 3.4 million insured individuals. The individual insurance histories contain precise information on health outcomes as well as information on income and mortality. Since the insurance fees depend on the income of the insured, individual income is recorded in the data. As in previous studies [e.g (5, 14, 21–23)], the external criterion of average income from salaries in Germany was used, which allows income to be grouped independently of the income structure within the data. This approach is particularly useful as the income structure in statutory health insurance data usually differs from that of the total population, as individuals with particularly high income are underrepresented in the data (24). Inflation adjustment underlines the resource-oriented approach, as purchasing power is kept constant over time. This approach allows for comparability with previous studies on social inequalities in morbidity and mortality [e.g (14, 21–23)]. The data used for this study are comparable with the German population in terms of age distribution and sex ratio. It has also been shown that the insurance population does not differ from the general population with respect to the distribution of employees subject to social insurance contributions. However, individuals insured with the AOKN are more likely to have occupations associated with lower socioeconomic status than the average population (24). Due to this, the total number of CFLE may be lower than in the general population. Since the data are limited to Lower Saxony, the results cannot be directly transferred to other federal states, especially again due to differing socioeconomic structures between the populations. However, we minimised the potential effect of differing social distributions by estimating all models either stratified or controlled for income.

Since we aimed to study trends in incidence over a wide age interval, it was not possible to use socioeconomic indicators other than income (e.g. education and occupational status) to depict social inequalities in CFLE, as in the insurance data this information is mainly available for the working-age population. Furthermore, the analyses were restricted to individual income. Since the data do not contain information on household composition, household income could not be calculated. This may have affected the findings especially in women, since the gender pay gap is still substantial in Germany with women having lower income (49). Furthermore, the share of part-time or precariously employed and of non-working population is higher among women than in men (54). Therefore, the level of individual income among women may be lower than compared to their household income, which may led to an underestimating of the extent of income inequalities in incidence and CFLE in women. Shi et al. could show that the level of income inequalities in life expectancy is to some extent also influenced by the definition of income (55). In accordance with this, previous analyses have shown that while the total level of income inequality tends to vary, both household income and individual income are largely reliable to measure health inequalities (56). However, since it cannot be ruled out that income inequalities have been underestimated to some extent, the results for women may be interpreted with some caution.

## Conclusion

This study reveals that incidence rates of total cancer as well as most site-specific cancers declined over time and led to strong increases in CFLE over time, irrespective of sex and income group. The results show that there are substantial income inequalities in CFLE which remained largely stable throughout the study period. Moreover, there are also clear differences with respect to cancer site. Successes have been achieved in the area of prevention in the past, as can be seen, for example, in the declining smoking rates in men in Germany. Although we could not measure this direct association, it can be assumed that this reduction in risk factors in the past had a positive effect on the development of incidence rates as seen for almost all cancer types considered in this study. Nevertheless, declining numbers of CFLE in skin cancer and female lung and cervical cancer, as well as the persisting income inequalities in cancer incidence should give pause for thought. Increased public health awareness and specific interventions for vulnerable subgroups should be strengthened to further reduce the incidence of and social inequalities in cancer.

## Data Availability Statement

The data analysed in this study are not freely publicly available due to protection of data privacy of the insured individuals by the AOK Niedersachsen (AOKN-Statutory Local Health Insurance of Lower Saxony). The data underlying this study belong to the AOKN and are therefore only available on request. Interested researchers can send data access requests to Jona Stahmeyer at the AOKN using the following e-mail address: Jona.Stahmeyer@aok.nds.de.

## Author Contributions

FT and JT developed the idea and research questions of the study. FT analysed the data and wrote the first draft of the manuscript. JE and JT were major contributors to the final manuscript. JH, JE, SG, HG, and JT contributed to the conception and discussion of the study and reviewed the work critically. All authors read, edited, and approved the final version of the manuscript.

## Funding

JE’s and JT’s work is supported by the AOK Niedersachsen (Statutory Local Health Insurance of Lower Saxony) as part of an ongoing project on morbidity compression. FT’s work is supported by the German Research Foundation (DFG) [grant number: TE 1395/1-1]. The funder was not involved in the study design, collection, analysis, interpretation of data, the writing of this article or the decision to submit it for publication.

## Conflict of Interest

The authors declare that the research was conducted in the absence of any commercial or financial relationships that could be construed as a potential conflict of interest.

## Publisher’s Note

All claims expressed in this article are solely those of the authors and do not necessarily represent those of their affiliated organizations, or those of the publisher, the editors and the reviewers. Any product that may be evaluated in this article, or claim that may be made by its manufacturer, is not guaranteed or endorsed by the publisher.

## Abbreviations

AOKN, Allgemeine Ortskrankenkasse Niedersachsen; CFLE, cancer-free life expectancy; GAI, German average income from salaries; HPV, human papillomaviruses; ICD-10GM, international statistical classification of diseases and related health problems, 10^th^ Revision, German Modification; SGB VI, book VI of the Social Code.
