# Supplementary material for: Time Trends and Income Inequalities in Cancer Incidence and Cancer-Free Life Expectancy – a Cancer Site-Specific Analysis of German Health Insurance Data
Source: Front Oncol. 2022 Apr 14;12:827028. doi: 10.3389/fonc.2022.827028 (PMC9046985; doi:10.3389/fonc.2022.827028)

Supplemental Material

Time trends and income inequalities in cancer incidence and cancer-free life expectancy – a cancer site-specific analysis of German health insurance data

Fabian Tetzlaff[1]*, Jens Hoebel[2], Jelena Epping[1,3], Siegfried Geyer[1,3], Heiko Golpon[3,4], Juliane Tetzlaff[1]

[1] Medical Sociology Unit, Hannover Medical School, Hanover, Germany

[2] Division of Social Determinants of Health, Robert Koch Institute, Berlin, Germany

[3] Comprehensive Cancer Center Hannover, Hannover Medical School, Hanover, Germany

[4] Department of Pneumology, Hannover Medical School, Hanover, Germany

*Corresponding author

E-mail: TetzlaffF@rki.de

Fig. S1 Time trend in age-specific total cancer incidence per 100,000 person-years by sex and income group

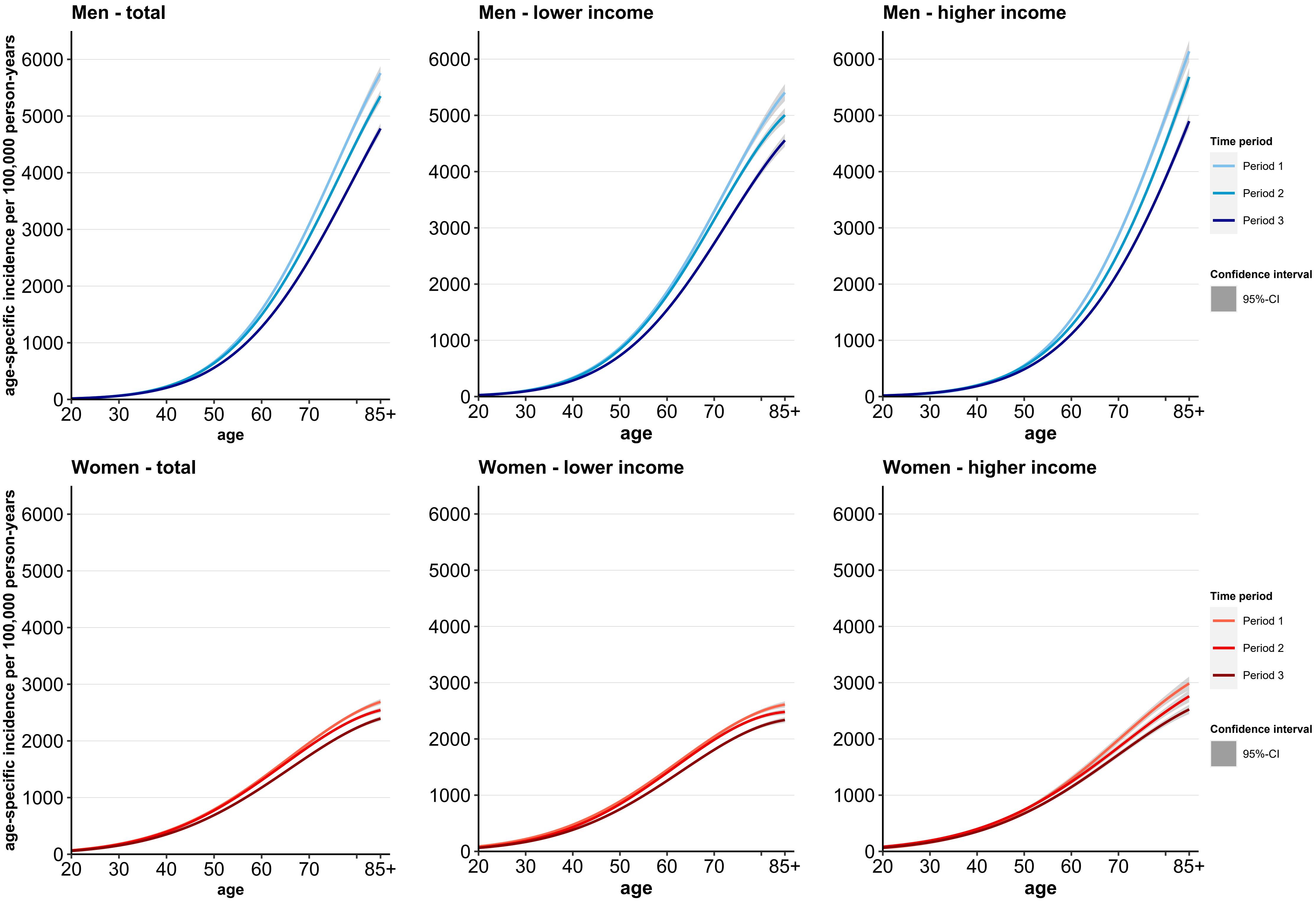

Fig. S2 Time trend in age-specific colon cancer incidence per 100,000 person-years by sex and income group

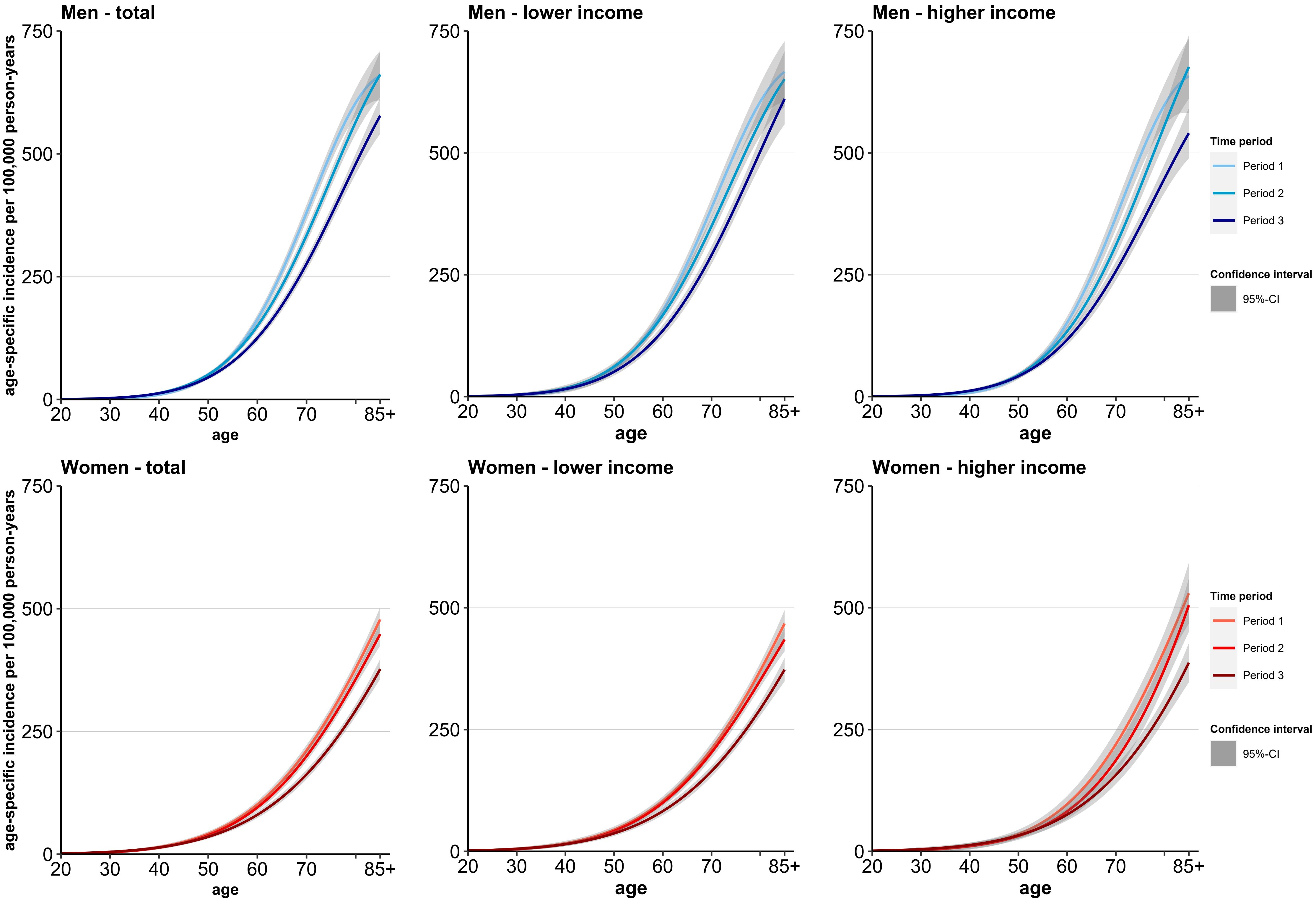

Fig. S3 Time trend in age-specific lung cancer incidence per 100,000 person-years by sex and income group

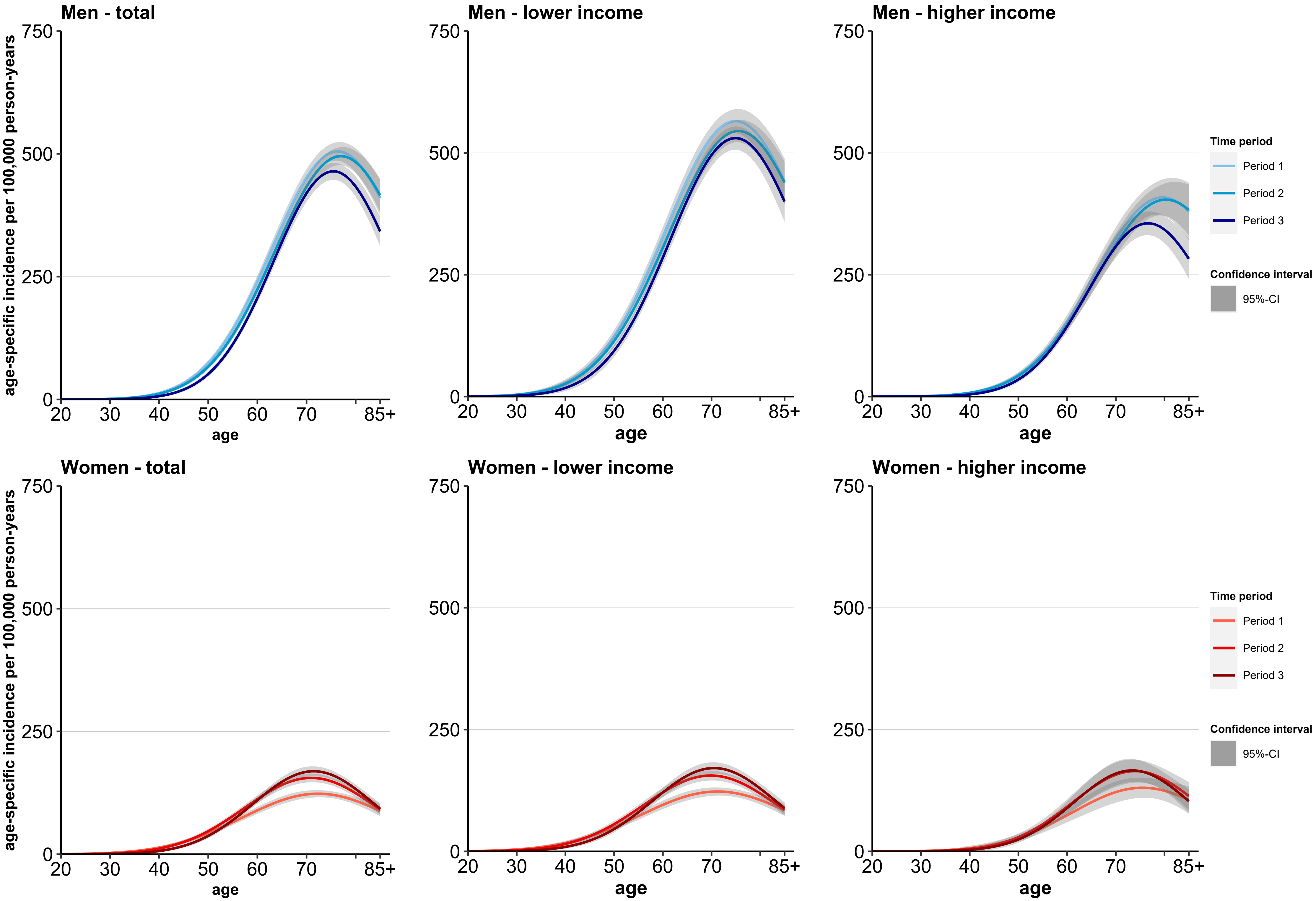

Fig. S4 Time trend in age-specific stomach cancer incidence per 100,000 person-years by sex and income group

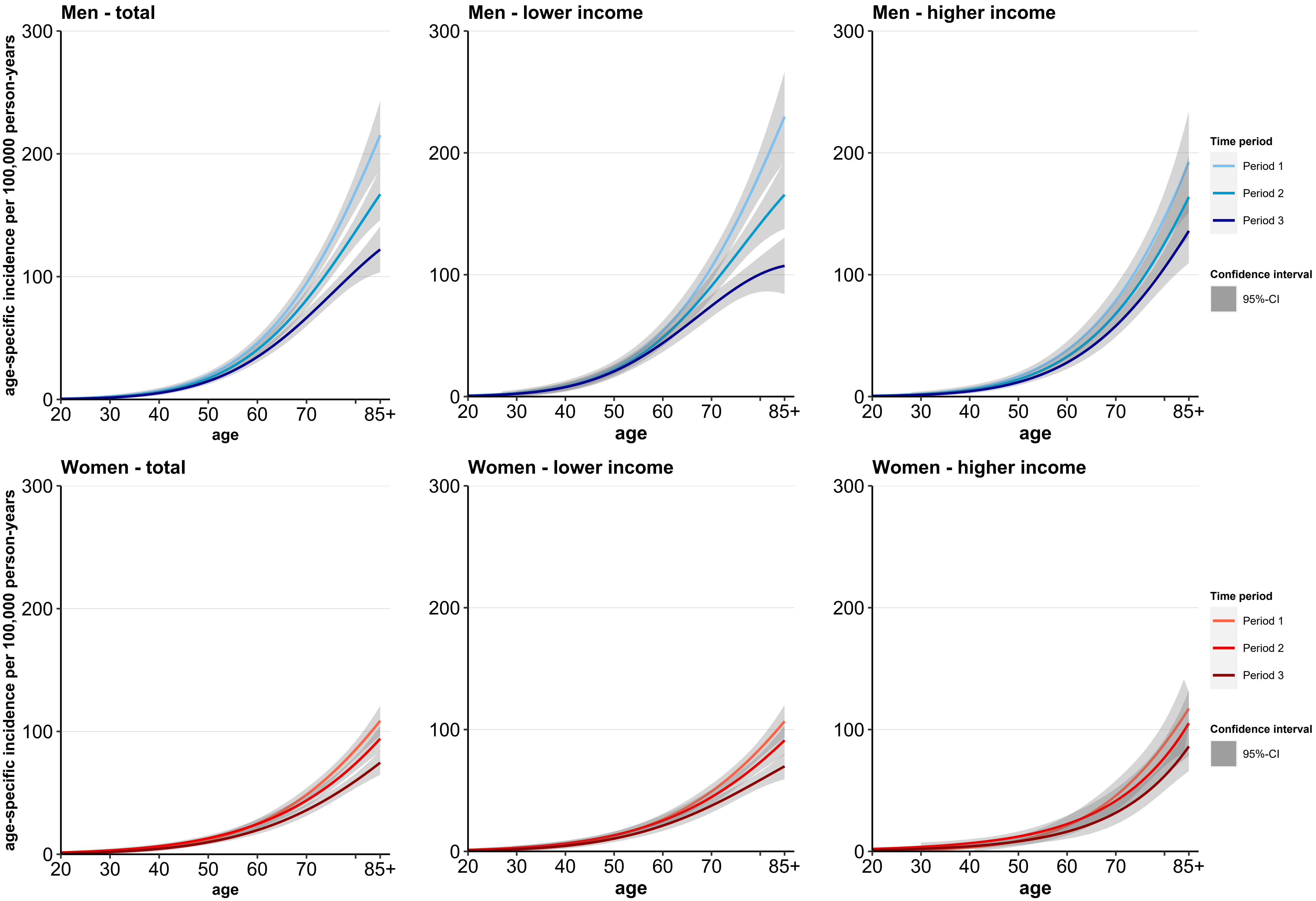

Fig. S5 Time trend in age-specific skin cancer incidence per 100,000 person-years by sex and income group

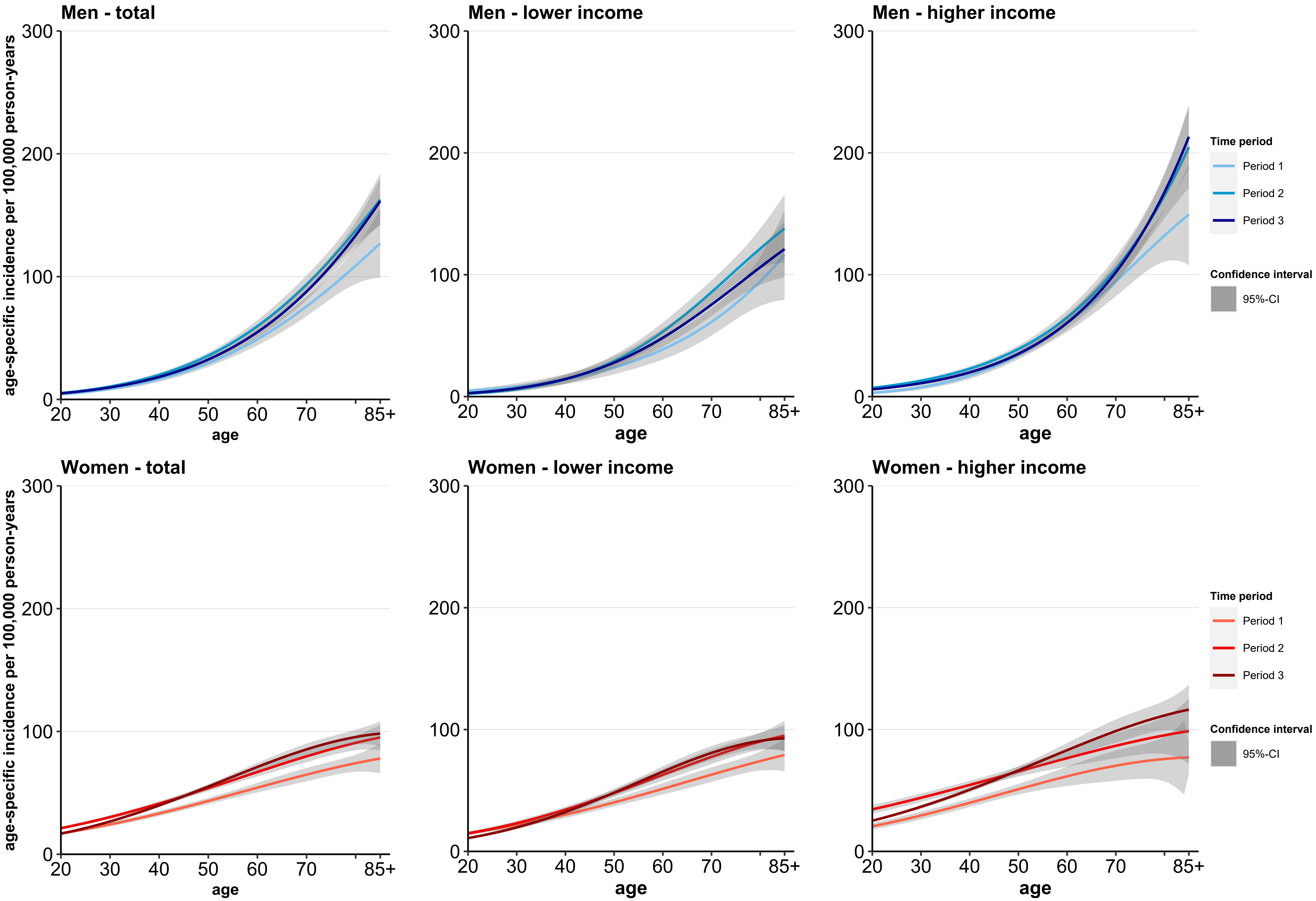

Fig. S6 Time trend in age-specific incidence (prostate cancer, breast cancer, and cervix uteri) per 100,000 person-years by sex and income group

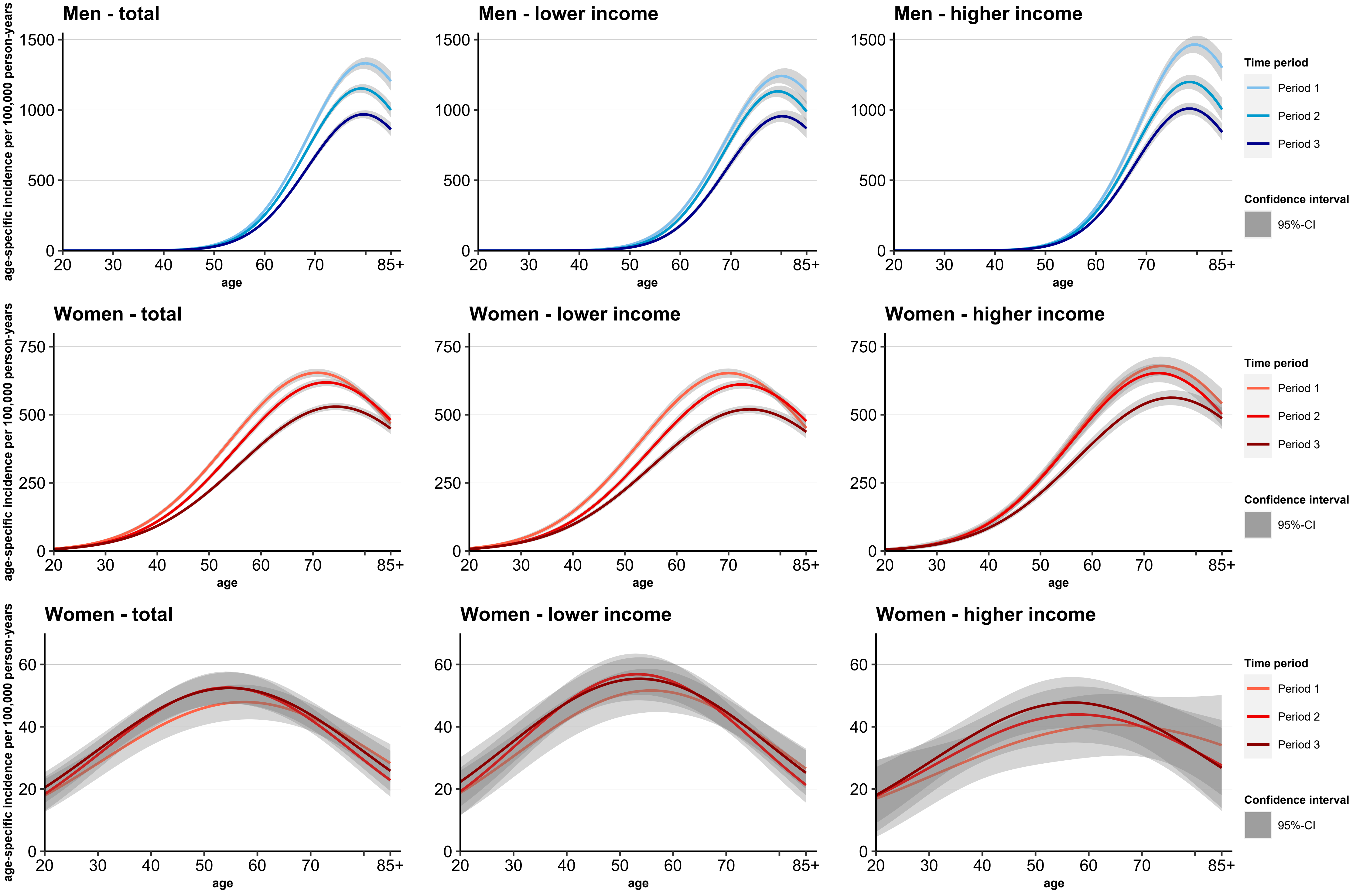

Fig. S7 Time trend in age-specific other cancer incidence per 100,000 person-years by sex and income group

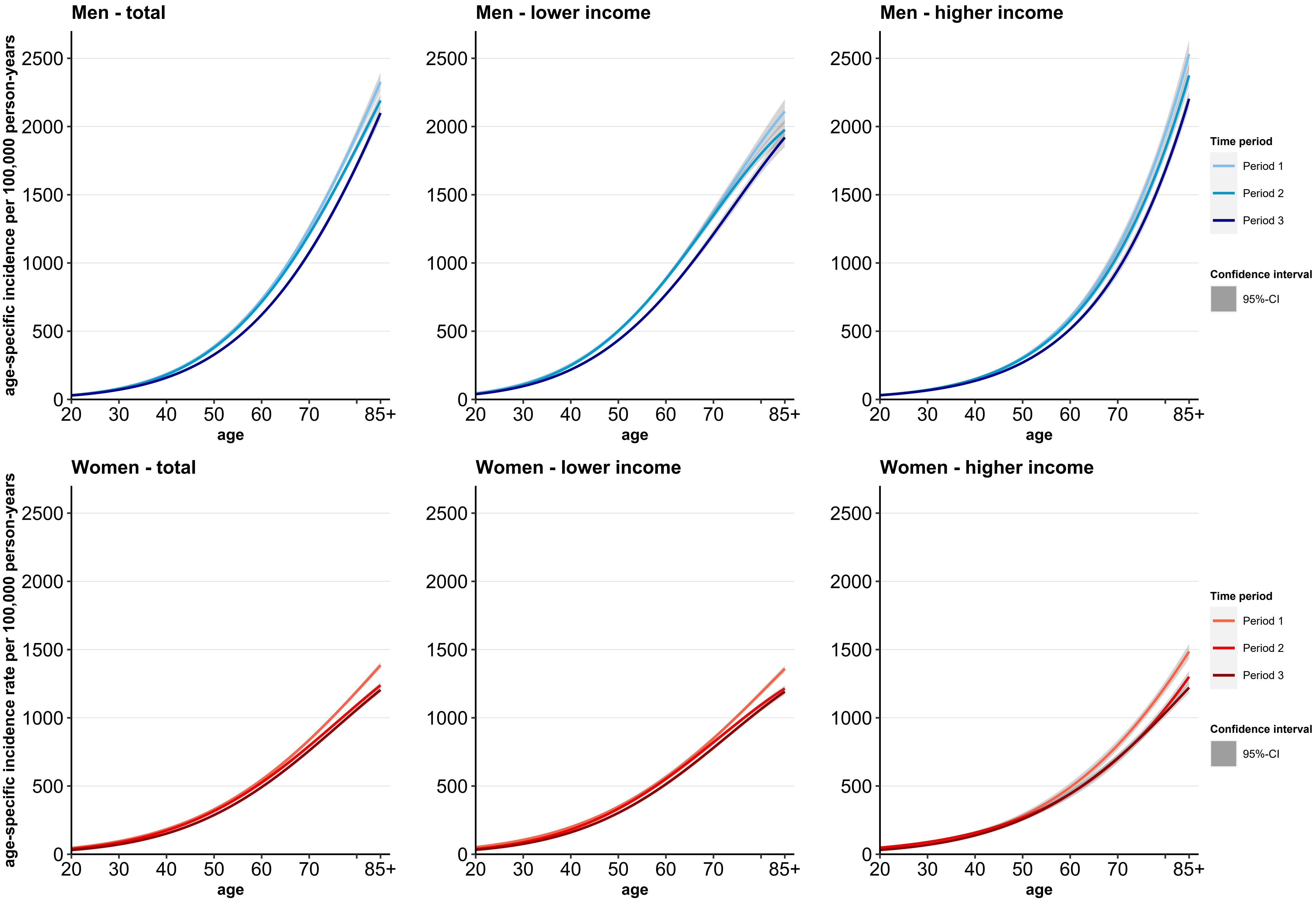

Fig. S8 Time trend in income inequality in cancer risk in men and women

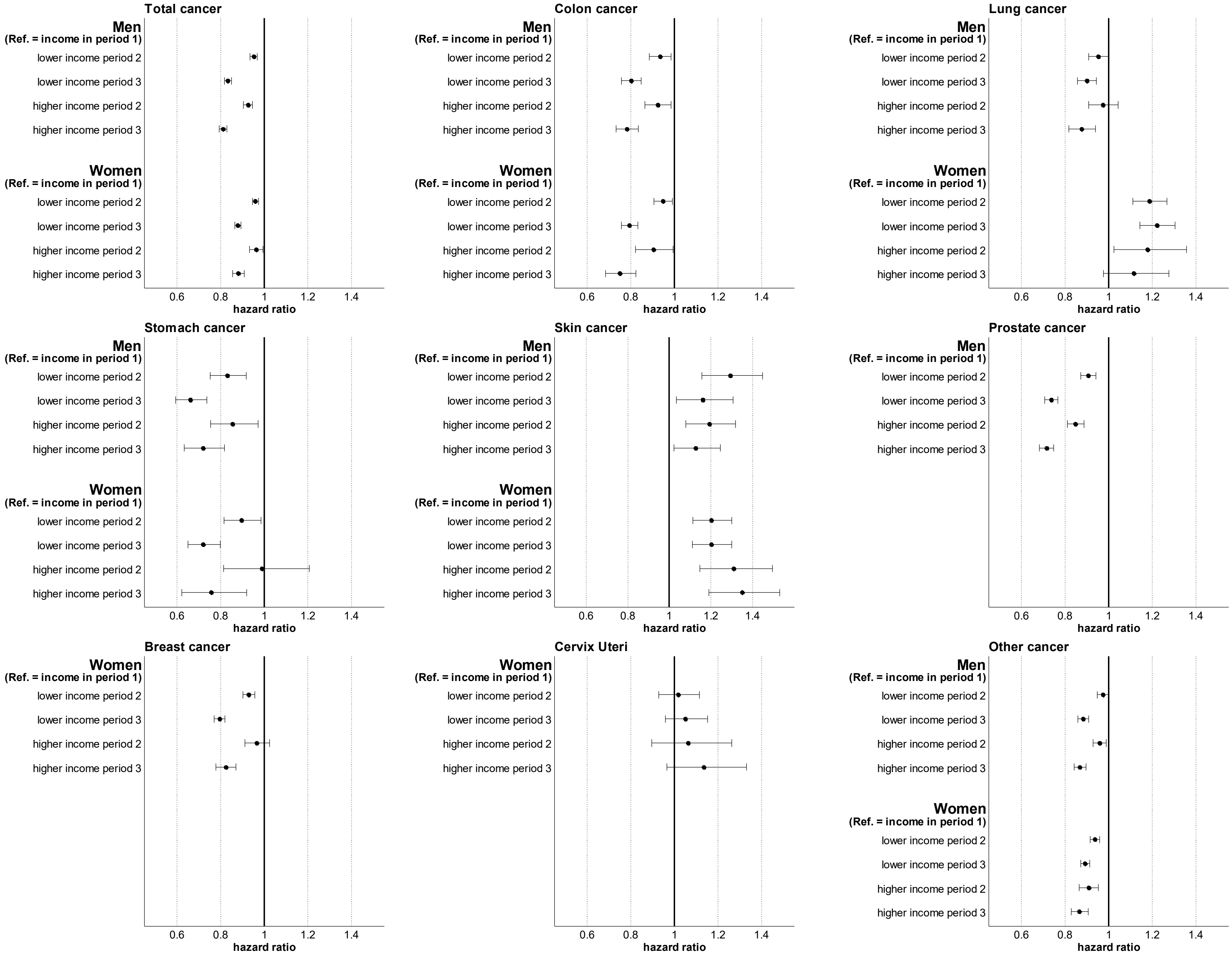

Note: All models were stratified for sex and income group, and are controlled for age (in single-year age groups as second-degree polynomial) and period, and were performed separately for total cancer and the single-cancer sites

Fig. S9 Time trend in cancer-free life expectancy in total cancer by sex and income group

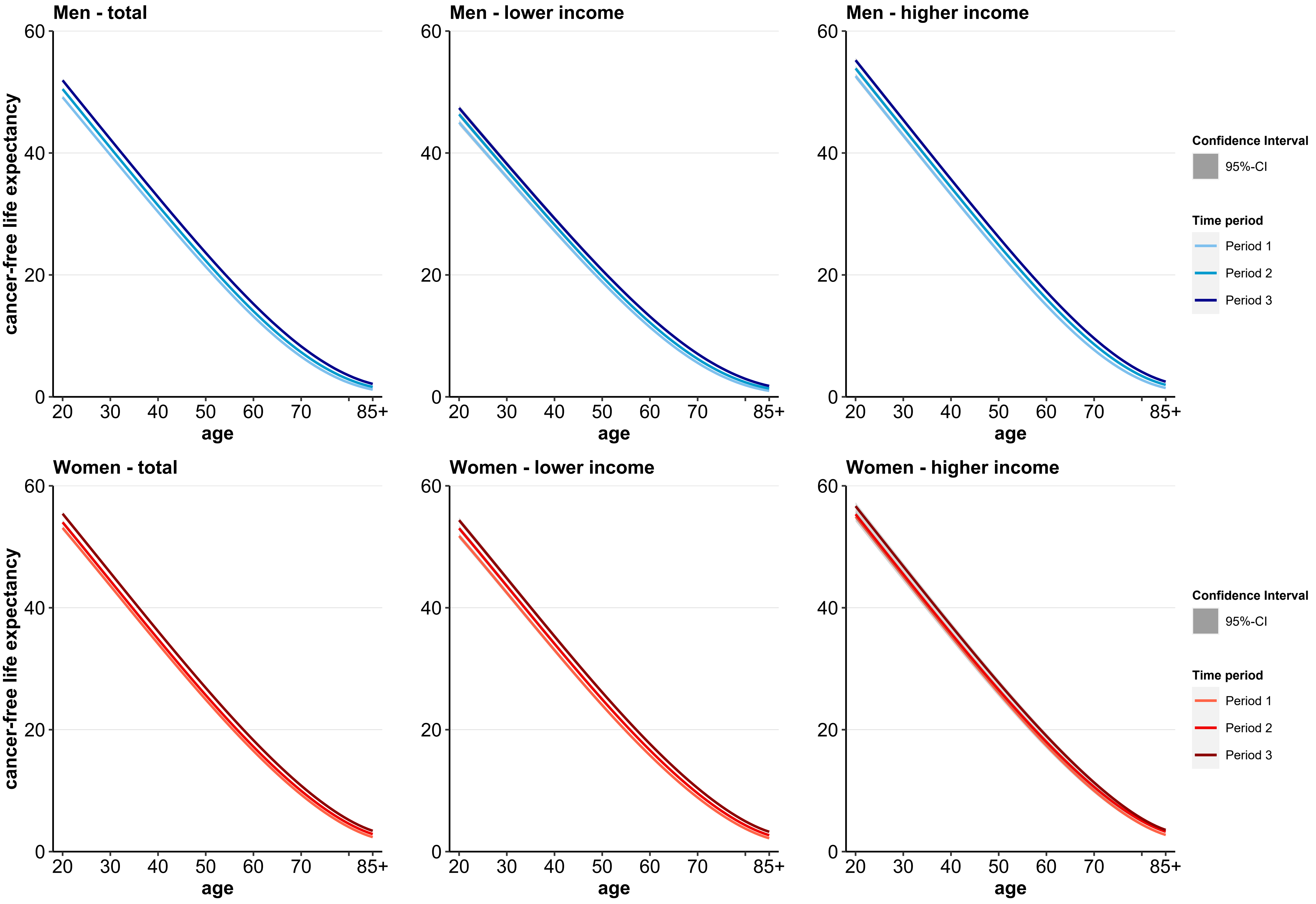

Fig. S10 Time trend in cancer-free life expectancy in colon cancer by sex and income group

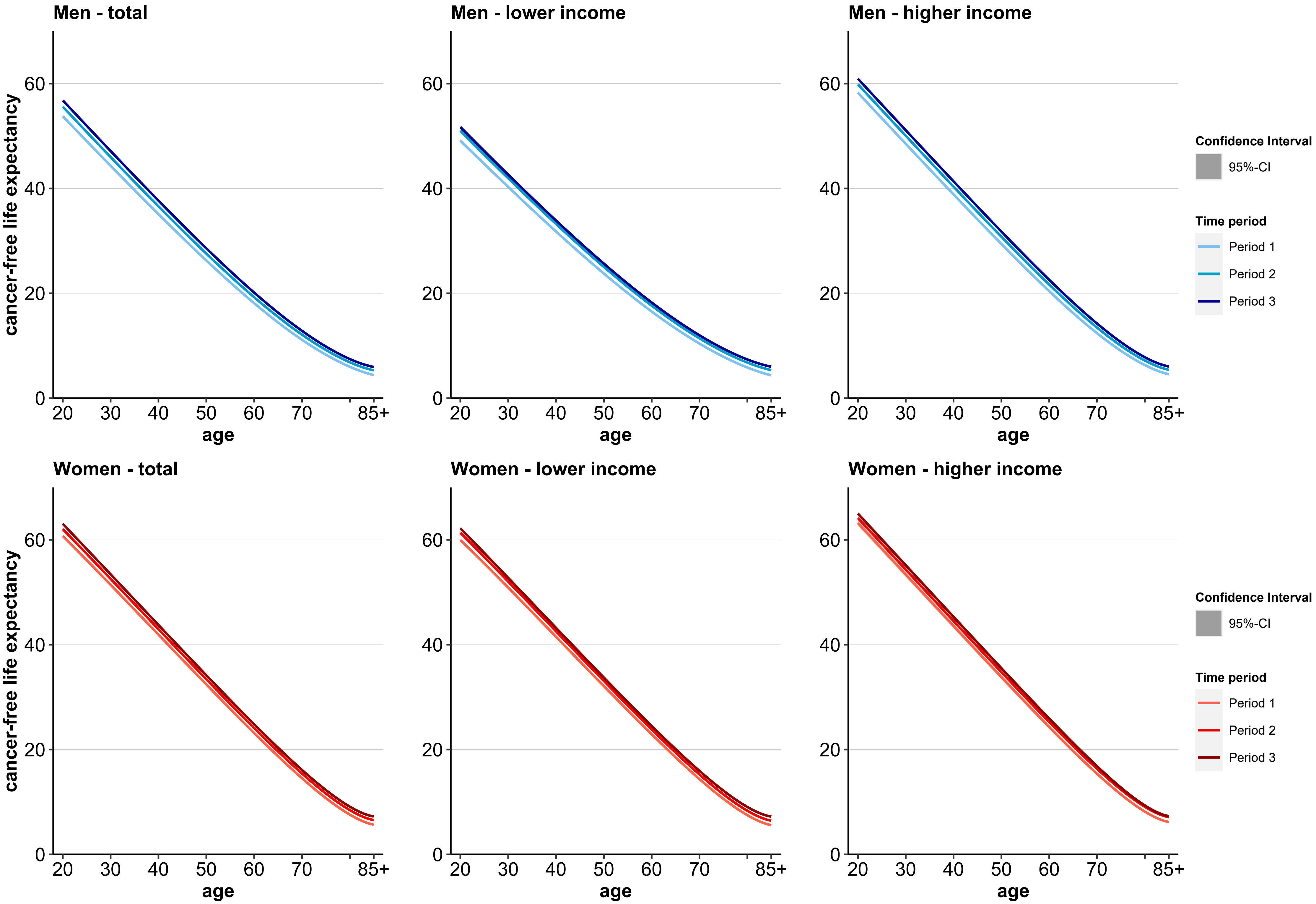

Fig. S11 Time trend in cancer-free life expectancy in lung cancer by sex and income group

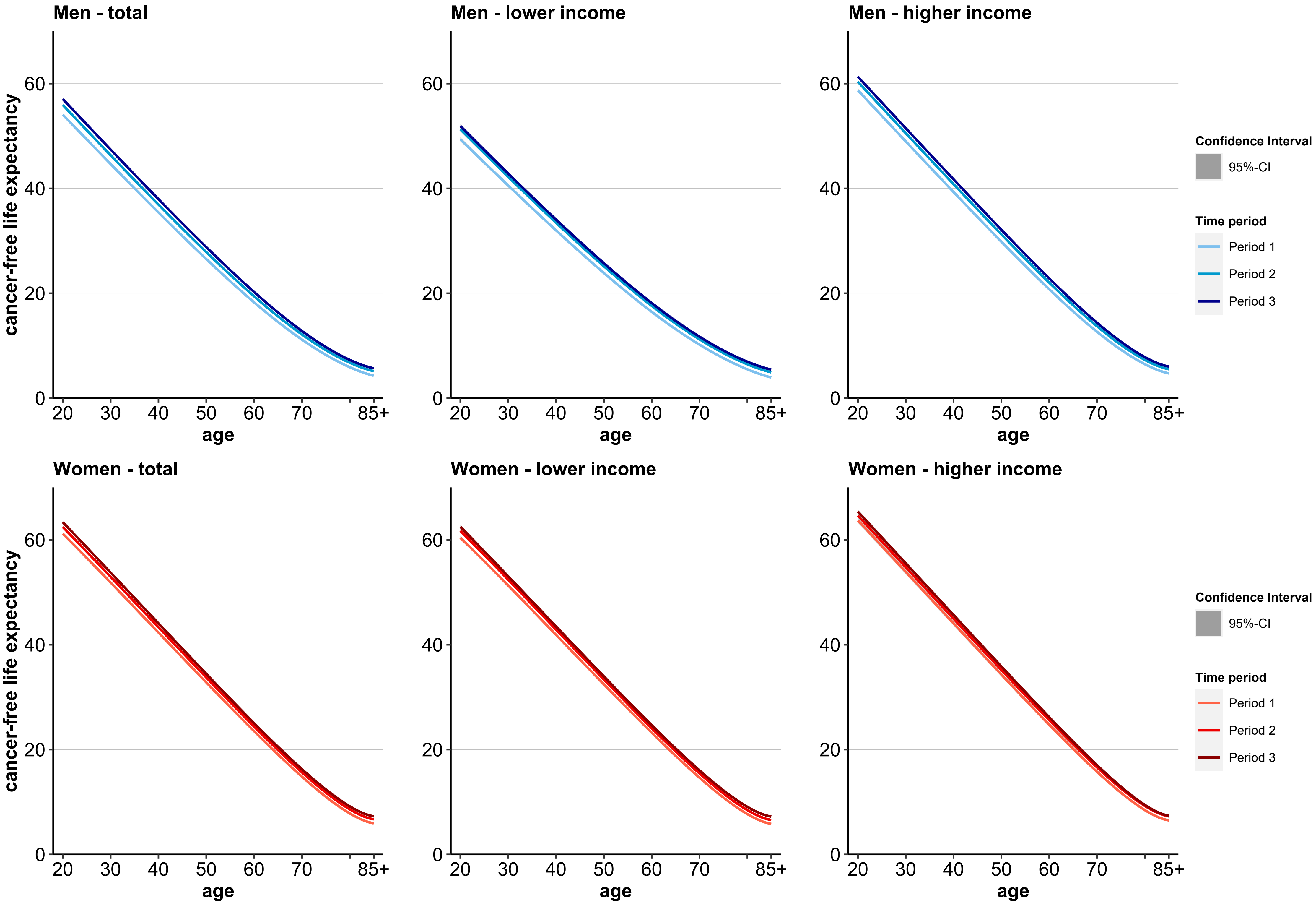

Fig S12 Time trend in cancer-free life expectancy in stomach cancer by sex and income group

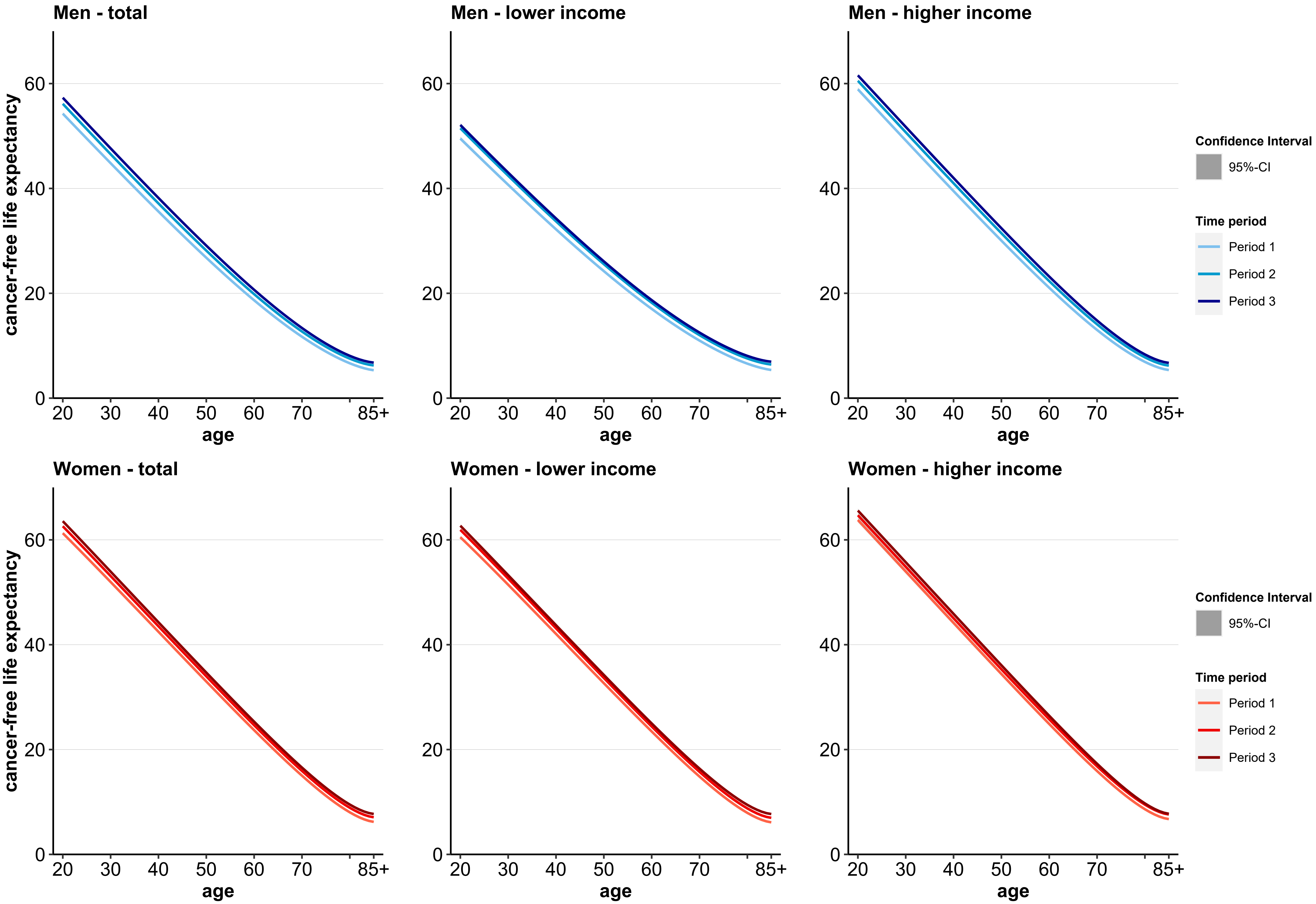

Fig. S13 Time trend in cancer-free life expectancy in skin cancer by sex and income group

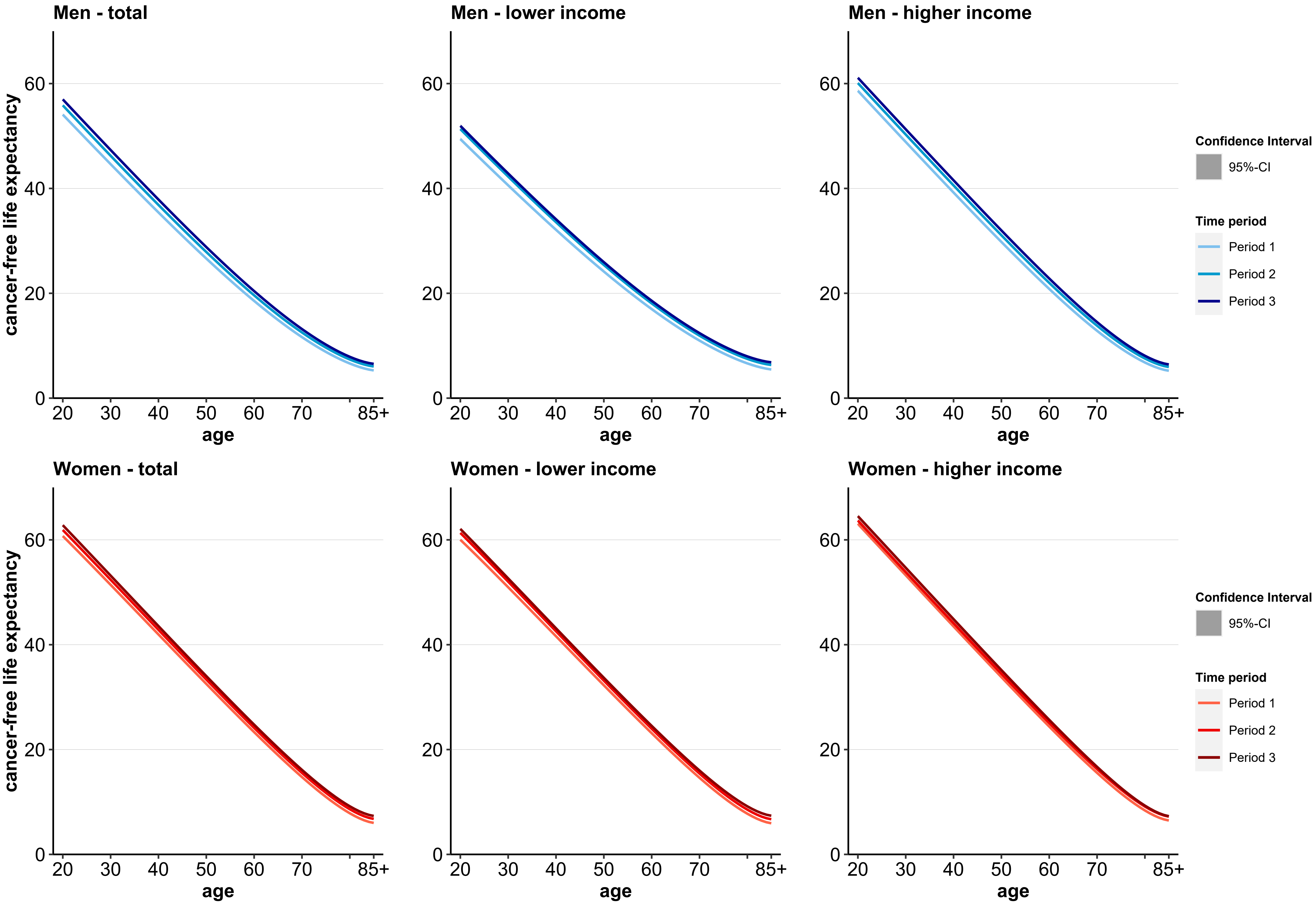

Fig. S14 Time trend in cancer-free life expectancy in prostate, breast, and cervix cancer by sex and income group

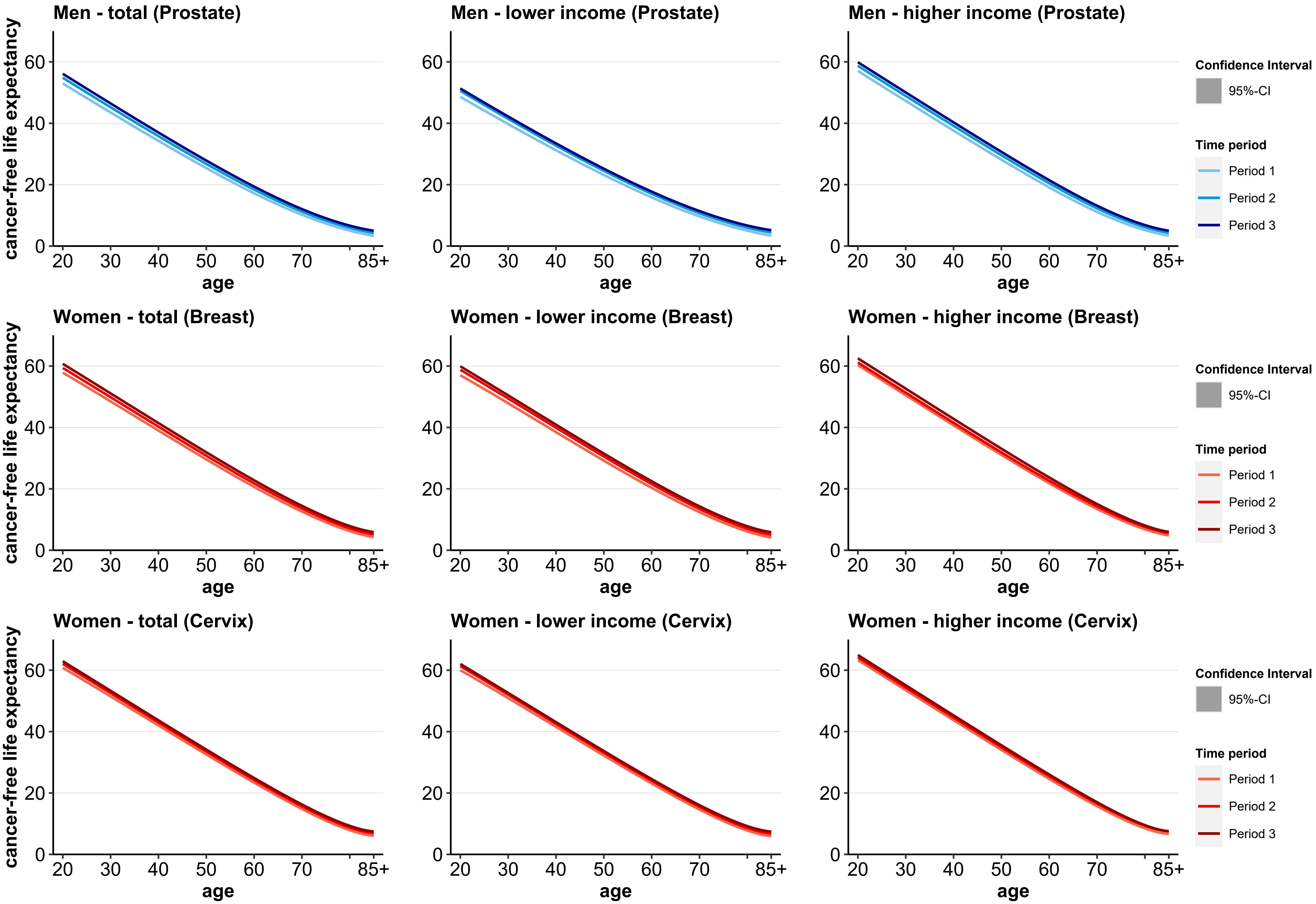

Fig. S15 Time trend in cancer-free life expectancy in other cancer by sex and income group

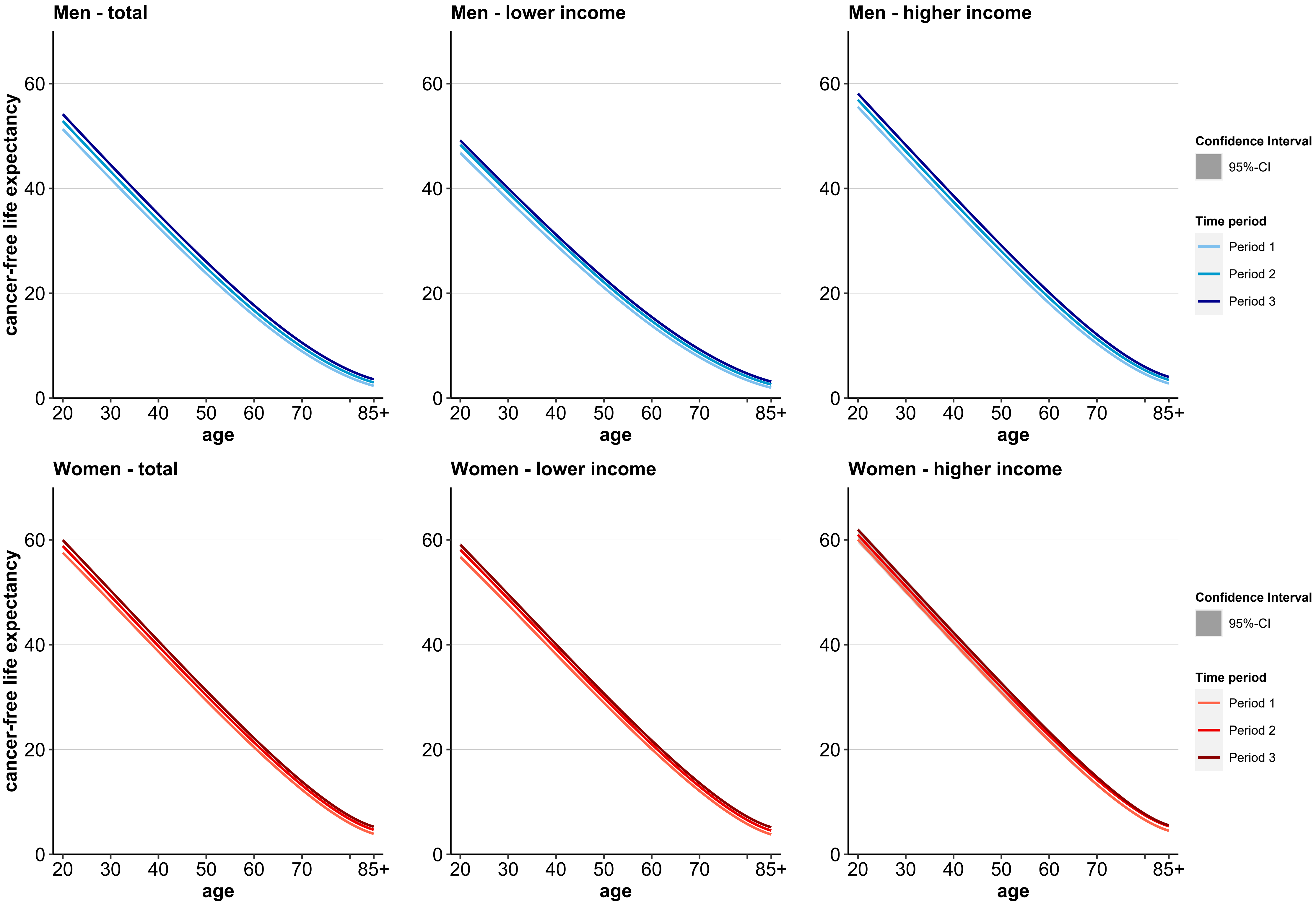

Supplement: Supplementary file 1 [file DataSheet_1.pdf]
